# Evaluating a Behavioural Theory-Based Board Game (S-S-LIBOG) Against Traditional Health Talk (HT) in Prostate Cancer Education: Findings from a Quasi-Experimental Study, Plus Introducing 17 Other S-S-LIBOGs †

**DOI:** 10.3390/healthcare13233135

**Published:** 2025-12-02

**Authors:** Frank Obeng, Mohammed Fadil, Aishah Fadila Adamu, Daniel Senanu Dadee-Seshie, Eric Nii Okai, Godson Agbeteti, Sylvester Appiah Boakye, Banabas Kpankyaano, Evans Kwaku Zikpi, Appiateng Wofa Boadu, Joyce Naa Aklerh Okai, Selasie Owiafe, Millicent Ofori Boateng

**Affiliations:** 1 Department of Surgery, Urology Unit, University of Health and Allied Sciences (UHAS), Ho PMB 31, Ghana; phadheelmuhammad@yahoo.com (M.F.); 2017aadamu@uhas.edu.gh (A.F.A.); danielsenanuseshie1@gmail.com (D.S.D.-S.); eniiokai18@som.uhas.edu.gh (E.N.O.); godsonagbeteti1@gmail.com (G.A.); appiahsylvester080@gmail.com (S.A.B.); kpankyaanob@gmail.com (B.K.); evanszikpi@gmail.com (E.K.Z.); davidappiateng22@gmail.com (A.W.B.); sowiafe19@som.uhas.edu.gh (S.O.); 2 Department of Medicine, Accra College of Medicine, Accra P.O. Box CT9828, Ghana; joycenaaokai@gmail.com; 3 Community Health Department, Ensign Global College, Kpong P.O. Box AK 136, Ghana; millicent.boateng@ensign.edu.gh

**Keywords:** prostate cancer literacy, public health educational interventions, interactive learning, behavior-change communication, analogue and digital versions of the game, comparison with health talks, quasi-experimental study, Ghana, gender disparities, knowledge, attitudes, perceptions, early detection

## Abstract

**Background:** Prostate cancer is a major public health concern in Ghana, where most cases present late and mortality remains high. Community education is essential for improving awareness and early detection. Traditional health talks are widely used, but interactive approaches such as board games have received little evaluation. **Aim:** To compare the effectiveness of a Social Cognitive Theory–Socioecological Model-based literacy board game (S-S-LIBOG) with a traditional health talk in improving prostate cancer knowledge, attitudes, and perceptions. **Methods:** A quasi-experimental, two-arm interventional study was conducted in a semi-urban Ghanaian cohort. Participants (n = 197) were allocated to either the board game arm (n = 80) or the health talk arm (n = 61) after accounting for attrition. A structured questionnaire measured knowledge, attitudes, and perceptions (KAP) before and after intervention. Statistical analyses at 5% alpha level included chi-square tests, two-proportion Z-tests, Wilcoxon signed-rank tests, and multivariate logistic regression. **Results:** Among participants, 29.4% were female, 64.5% male, and 6.1% other genders. Tertiary education was reported by 81.7%, secondary 9.6%, postgraduate 5.6%, and primary 3.0%. Ethnicities: Ewe 41.6%, Akan 26.9%, Northern 13.7%, Ga 6.6%, Guan 1.5%, others 9.6%. Rural dwellers: 29.9%. LIBOG improved ‘good knowledge level’ from 35.0% at baseline to 60.0% post-intervention, compared to 35.0% to 62.3% by the Health Talk (HT). S-S-LIBOG also narrowed gender, education, and lifestyle disparities in KAP, with males showing higher odds of positive attitude (OR = 4.16, *p* = 0.004) and perception (OR = 2.79, *p* = 0.047), and rural residents having increased odds of good knowledge (OR = 4.39, *p* = 0.041) post—its intervention. HT similarly equalized disparities, except for perception, which remained linked to education. The significant improvements in knowledge were (LIBOG: z = 2.85, *p* = 0.004; HT: z = 3.10, *p* = 0.002). Even though health talks achieved higher overall knowledge gains, no statistically significant difference in overall effectiveness was observed between the two methods (Wilcoxon W = 102.0, *p* = 0.107). Acceptability of the board game was high, with over 80% of participants reporting satisfaction. **Conclusions:** The S-S-LIBOG board game was not inferior to the traditional health talk, showing particular strengths in enhancing attitudes and perceptions. Its interactive and culturally adapted design makes it a feasible adjunct to conventional health education methods. Future studies should examine long-term impacts and application in more diverse populations. This study was retrospectively registered by the Pan African Clinical Trial Registry on 10 October 2025; with the Trial Registration number PACTR202510512711680.

## 1. Introduction

Prostate cancer (PCa) remains a major public health challenge globally, both in incidence and mortality. It is consistently ranked among the top five cancers worldwide, following lung, breast, and colorectal cancer in incidence, and trailing only lung, liver, and stomach cancers in mortality [1,2,3,4,5]. Data from the Global Burden of Disease (GBD) study confirm that PCa is the most commonly diagnosed cancer among men [3,4,5,6,7,8]. Despite advances in early detection and treatment in high-income countries, wide disparities persist. While incidence and mortality rates have stabilized or declined in many developed settings due to organized screening and access to curative treatment, late-stage presentation and high mortality continue to characterize low- and middle-income countries (LMICs), especially in sub-Saharan Africa [4,6,7].

In Africa, the prostate cancer burden is amplified by weak cancer registration systems, inadequate screening programs, and sociocultural barriers to health-seeking behavior [6,7,8]. The proportion of global cancer cases occurring in LMICs rose from 15% in 1970 to 56% in 2008 and is projected to reach 70% by 2030 [4,5,6,7,8,9]. For PCa, these trends translate into an increasing number of men being diagnosed late, often when curative options are no longer viable and palliative care becomes the mainstay [9,10,11,12,13,14,15]. Black men, both within Africa and the diaspora, face disproportionately higher risks of incidence, advanced disease at diagnosis, and mortality, underscoring the need for context-specific interventions [10,11,12,13,14,15,16,17].

In Ghana, prostate cancer is the leading male malignancy and carries one of the highest mortality rates in the world. Studies report that up to 80% of Ghanaian men with PCa present at advanced stages, resulting in a case fatality rate of about 55% [9,13,14,18]. Awareness and knowledge levels remain suboptimal: while community surveys suggest relatively high awareness (85.8%), adequate knowledge is seen in only half of respondents, and actual screening uptake is very low (10.2%) [13,15,16,17,18,19]. Barriers include lack of health education, cultural stigma, misconceptions about screening, and fear of diagnosis [14,15,16,17,18,19,20,21]. These factors reinforce the urgent need for innovative educational strategies that can engage communities more effectively than conventional didactic health talks.

Traditional health education approaches—including group talks, posters, and recorded media—remain the most common strategies in Ghana [13,17,18,19,20,21,22]. While effective to some extent, their capacity to achieve lasting behavioral change has been modest [18,19,20,21,22,23]. Emerging evidence suggests that participatory, interactive methods may be more impactful [18,20,21,22,23,24,25,26,27,28,29]. Gamified educational interventions, such as board games, leverage social learning, peer influence, and experiential engagement to improve retention and encourage positive health behaviors [18,20,21,22,23,24,25,26,27]. Models like the Social Cognitive Theory (SCT) and the Socioecological Model (SEM) provide theoretical grounding, emphasizing the interplay between individual cognition, social reinforcement, and environmental context in shaping health behaviors [22,23,24,25,26,27,28,29]. Previous applications of board games for health education—including HIV/AIDS, malaria, and urological training—have shown promise in enhancing knowledge, engagement, and satisfaction compared with traditional lectures [20,21,22,23,24,25,26,27,28,29,30].

Rationale and Objective: Given the disproportionate burden of prostate cancer in Ghana, the limited effectiveness of traditional health talks, and the emerging promise of interactive learning approaches, this study introduces and evaluates a novel SCT–SEM-based literacy board game (S-S-LIBOG, or LIBOG for shorter). The objective was to compare its effectiveness against the conventional health talk (HT) in improving knowledge, attitudes, and perceptions about prostate cancer among a semi-urban Ghanaian population.

The secondary objective was to evaluate the usability of the board game amongst participants, in that arm of the study.

Hypothesis: The study’s null hypotheses were:

There is no difference in the proportion of participants with good knowledge of prostate cancer before and after a standard health talk intervention.

There is no difference in the proportion of participants with good knowledge of prostate cancer before and after the session of interaction with the LIBOG game.

There is no difference in the median percentage gains between LIBOG and Health Talk interventional groups in the study.

Previous Presentation: This work was presented as a Poster during the University of Health and Allied Sciences (Ho, Ghana) Research Conference held in October, 2024. It was also accepted as a conference abstract at the 2025 International Meeting on Simulation in Health Conference, held in January 2025 in Orlando USA, (the Authors could not attend this conference though). The primordial beginnings of the work were also posted as a pre-print on ResearchGate in July 2024 [22], and is available on the following link: https://www.researchgate.net/publication/382708683_Evaluating_the_Effectiveness_of_a_Socio-Ecological_Model-Based_Snake-and-Ladder_Board_Game_Versus_Traditional_Educational_Methods_in_Prostate_Cancer_Awareness_and_Education_A_study_in_Southeastern_Gha? (accessed on 19 October 2025).

## 2. Materials & Methods

### 2.1. Conceptual Framework

The Socio-Ecological Model (SEM) and Social Cognitive Theory (SCT) provided the theoretical foundation for this study. SEM highlights multiple levels of influence on health behavior (individual, interpersonal, community, and societal), while SCT emphasizes observational learning, reinforcement, and self-efficacy. Both models guided the design of the board game intervention. The detailed write-up on the conceptual framework is in Section S1.0 of the Supplementary Material document. Figures S1 and S2 in the Supplementary Material present the SEM and SCT conceptual framework diagrams as applied in this study.

#### 2.1.1. Study Design and Setting

This was a single-camp, quasi-experimental, two-arm comparative effectiveness study conducted in the Ho municipality, Ghana, between June and November 2024. The intervention compared two educational approaches for prostate cancer literacy: (i) a Social Cognitive Theory–Socioecological Model-based board game (S-S-LIBOG), and (ii) a conventional health talk. Pre- and post-intervention surveys were administered to assess changes in knowledge, attitudes, and perceptions (KAP).

This study was retrospectively registered by the Pan African Clinical Trial Registry on 10 October 2025; with the Trial Registration number PACTR202510512711680.

#### 2.1.2. Participants and Recruitment

Eligible participants were men and women [28] aged ≥12 years who could read, write, and comprehend either English or a local language, and who provided informed consent. Medical doctors and clinical-year medical students were excluded to minimize professional bias.

A finite population of approximately 320 individuals was identified from three purposively selected sites:

Ho Teaching Hospital urology outpatient clinic (patients and caregivers), University of Health and Allied Sciences School of Nursing and Midwifery (students), Asogli hostel facility housing pre-clinical medical students.

Proportional allocation ensured that recruitment reflected site representation. Participants were assigned to two groups using systematic random sampling followed by dice toss to determine intervention allocation.

#### 2.1.3. Sample Size Determination

The formula applied was the one for sample size calculation for comparing two independent proportions (two-sided Z-test, equal allocation by Fleiss et al., 2003) [31]. The sample size was estimated to detect an expected 20% improvement in knowledge (β = 0.20) [32]. This aligns with an 80% power (β = 0.20) and a 5% significance level, assuming equal allocation between arms. This yielded a minimum of 178 participants. To account for attrition, the target was increased to 200 (Please see the Supplementary Material (Section S2.2) for the full sample size calculation steps). Ultimately, 197 participants completed the baseline assessment.

**Figure 1:**
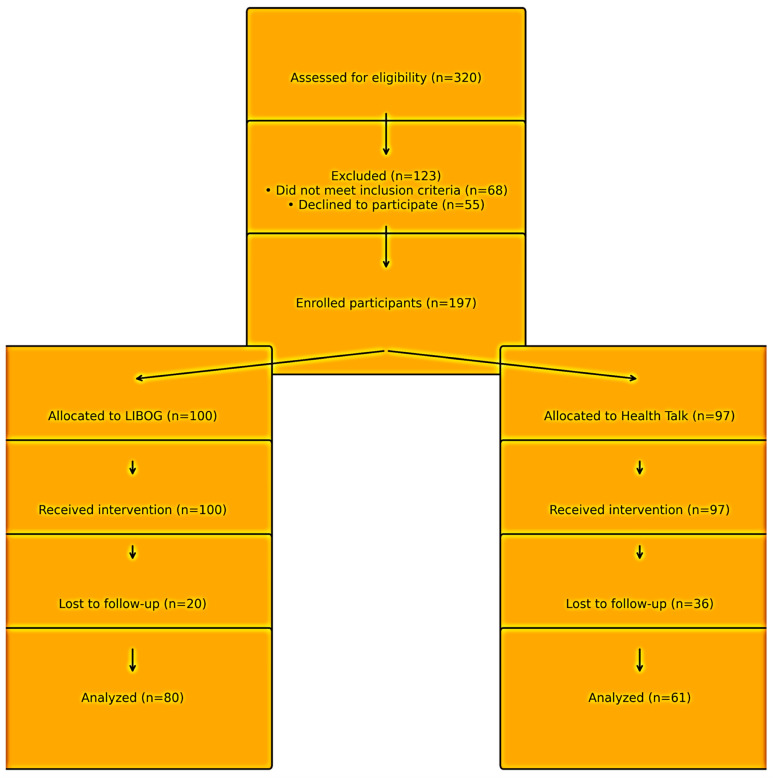
Flow of participants through the study. LEGEND: The diagram shows screening, enrollment, allocation, follow-up, and analysis for the two study arms. A total of 320 individuals were assessed for eligibility, of whom 123 were excluded (68 did not meet inclusion criteria; 55 declined to participate). One hundred ninety-seven participants were enrolled, with 100 allocated to the S-S-LIBOG (Social Cognitive Theory–Socioecological Model-based Literacy Board Game) arm and 97 allocated to the Health Talk (HT) arm. All allocated participants received the assigned intervention. Attrition occurred in both arms (LIBOG: 20; HT: 36), resulting in 80 and 61 participants, respectively, included in the final analysis. Abbreviations: LIBOG = Literacy Board Game; HT = Health Talk.

Sampling and Allocation: From the frame of 197 participants, systematic random sampling with k = 2 was employed (the starting point ‘n’ was also determined by the toss of a dice). This divided participants into two groups (1 and 2). Intervention assignment to groups was decided through repeated dice tosses conducted by a neutral arbiter. The group whose number appeared more frequently first was allocated to the LIBOG arm, and the other to the Health Talk arm. Finally, a total of 141 participants were available post-intervention (attrition rate: 28.4%) Figure 1.

#### 2.1.4. Intervention Arms

**Figure 2:**
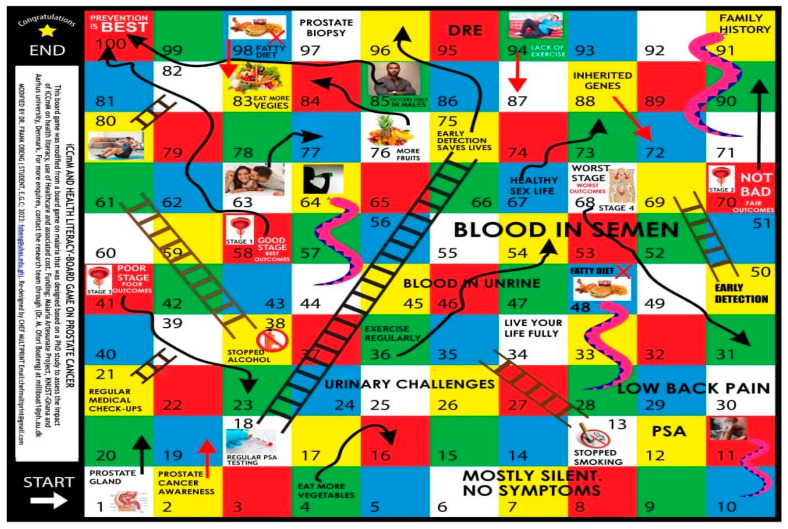
An attached image of the innovated SEM-SCT-based prostate cancer literacy board game (S-S-LiBoG). Legend: A snakes-ladders-and-arrows (Ludo) board game. Source: authors’ design, adaptation, and creation, 2023 to 2025: Obeng et al., 2024 [22]. (Note: The game is protected by a Copyright License (under the Republic of Ghana Copyright License Law: number xx0004726xx, x00-469/2024x). This was the health literacy tool for the study arm of this paper.

Board Game (S-S-LIBOG): A snakes-and-ladders style ludo game, modified with prostate cancer–related question cards and chance tiles, based on SCT and SEM principles. The game was co-designed with stakeholders and pilot-tested prior to implementation (Figure 2). A detailed description, rules, and hyperlinks to the game-packs are provided in Section S2.1 of the Supplementary Material.

Health Talk (Control): A standardized didactic lecture using PowerPoint slides, delivered by trained facilitators in the same community settings. The link to the full PowerPoint slides is available in Section S2.1 of the Supplementary Material document.

Allocation Concealment and Contamination Prevention: Group assignment was concealed using sealed opaque envelopes. Each intervention was held at different venues. Participants were briefed not to exchange materials or discuss intervention content, minimizing cross-group contamination.

### 2.2. Data Collection and Instruments

Data were collected using a structured knowledge, attitudes, and perceptions (KAP) questionnaire developed for this study. The tool, based on a 5-point Likert scale, covered sociodemographic characteristics, knowledge of risk factors, symptoms, treatment methods, attitudes toward screening, and perceptions of prostate cancer. Self-administered questionnaires were used for literate participants; interpreters assisted for low-literacy participants. A Post-Study System Usability Questionnaire (PSSUQ) assessed user experience with the board game. The Likert Scale-based questionnaires for the main study and the Post Usability Testing for the LIBOG is presented in Table A1, under the Appendix A of this paper.

#### 2.2.1. Outcome Measures

The primary outcomes were changes in KAP scores from pre- to post-intervention within each arm. Secondary outcomes included acceptability and satisfaction with the board game.

#### 2.2.2. Statistical Analysis

Data were entered in Microsoft Excel 2016 and analyzed in Stata version 17. Descriptive statistics summarized participant characteristics. Within-group changes in proportions were assessed using the two-proportion Z-test. Between-group differences in KAP score changes were analyzed using the Wilcoxon signed-rank test. Chi-square tests explored associations between demographic factors and KAP. Logistic regression identified independent predictors of knowledge, attitude, and perception outcomes. Statistical significance was set at *p* < 0.05.

#### 2.2.3. Ethical Considerations

The study was approved by the Ho Teaching Hospital Research Ethics Committee (HTH-REC (40) FC_2024; approval date: 26 November 2024). Written informed consent was obtained from all participants. For those with limited literacy, the consent form was read in the preferred local language, with verbal consent documented in the presence of an impartial witness. Confidentiality and anonymity were maintained throughout.

Trial Registration and Deviations: The study was retrospectively registered in the Pan African Clinical Trials Registry (PACTR202510512711680) on 10 October 2025. The delay was due to administrative timing between ethical clearance and registry acceptance. Nevertheless, all core procedures followed a time-stamped protocol archived with the principal investigator and ethics committee. The primary endpoints (knowledge, attitudes, and perceptions) remained unaltered. No deviation affected data collection or interpretation.

## 3. Results

### Sociodemographic Characteristics of Study Participants (Pre-Intervention)

A total of 197 participants completed baseline assessment, of whom 141 (71.6%) were retained for post-intervention analysis (attrition rate 28.4%). Gender was self-reported with three options: Male, Female, and Others. Males constituted 64.5% (n = 127), females 29.4% (n = 58), and 6.1% (n = 12) identified as other genders. The ‘Others’ category (6.1%) represented participants who did not identify within the binary gender categories. Due to its small cell size, it was retained descriptively but excluded from inferential analyses. The mean age was 34.3 years (SD ± 10.1; range 21–86), with the majority in the 31–50-year age group (58.4%). Most participants had tertiary education (81.7%), while smaller proportions reported secondary (9.6%), primary (3.0%), and postgraduate education (5.6%). Ethnically, 41.6% were Ewe, 26.9% Akan, 13.7% Northern Ghanaian, 6.6% Ga, 1.5% Guan, and 9.6% other. Nearly half resided in urban areas (46.2%), with 29.9% rural and 23.9% suburban. Lifestyle factors showed that 92.9% were non-smokers, 69.5% abstained from alcohol, and 71.1% reported sedentary-to-light exercise habits. Family history of prostate cancer was present in 4.6%, while 8.6% reported breast cancer, 2.5% ovarian cancer, 4.6% bladder cancer, and 3.0% gastrointestinal cancers in relatives. Baseline participant characteristics are summarized in Table 1.

Baseline Knowledge, Attitudes, and Perceptions (KAP), and Associations with Sociodemographic Factors:

Prior to intervention, 35.0% demonstrated good knowledge of prostate cancer, 54.4% expressed positive attitudes toward screening, and 41.1% showed positive perceptions, overall. Detailed baseline KAPs are presented in Table 2.

Associations at baseline:

Statistically significant associations were observed between sociodemographic and lifestyle characteristics and the KAPs. The associations are summarized below; (and the full details are found in Section S3.0 of the Supplementary Material document).

Knowledge: and education (χ^2^ = 13.50, *p* = 0.036), gender (χ^2^ = 19.92, *p* = 0.001), exercise (χ^2^ = 16.91, *p* = 0.010), diet (χ^2^ = 11.38, *p* = 0.023), tobacco use (χ^2^ = 11.90, *p* = 0.018), and alcohol use (χ^2^ = 19.84, *p* = 0.001).

Attitudes: and gender (χ^2^ = 32.60, *p* < 0.001), ethnicity (χ^2^ = 34.66, *p* < 0.001), education (χ^2^ = 27.40, *p* < 0.001), and lifestyle factors (exercise, tobacco, alcohol; all *p* < 0.01).

Perceptions: and gender (χ^2^ = 61.25, *p* < 0.001), ethnicity (χ^2^ = 37.18, *p* < 0.001), education (χ^2^ = 43.72, *p* < 0.001), and family cancer histories (ovarian, bladder, GIT cancers; all *p* < 0.05).

For predictors of KAP Outcomes at baseline, multivariate logistic regression analysis identified significant predictors:

Knowledge: regular exercise was the only independent predictor (AOR = 1.45, 95% CI: 1.06–1.98, *p* = 0.021).

Attitude: male gender (AOR = 2.56, 95% CI: 1.30–5.01, *p* = 0.006), higher income (AOR = 1.34, 95% CI: 1.01–1.77, *p* = 0.040), and exercise (AOR = 1.58, 95% CI: 1.15–2.17, *p* = 0.005) predicted positive attitudes.

Perception: male gender strongly predicted positive perception (AOR = 16.84, 95% CI: 4.92–57.63, *p* < 0.001).

**Figure 3:**
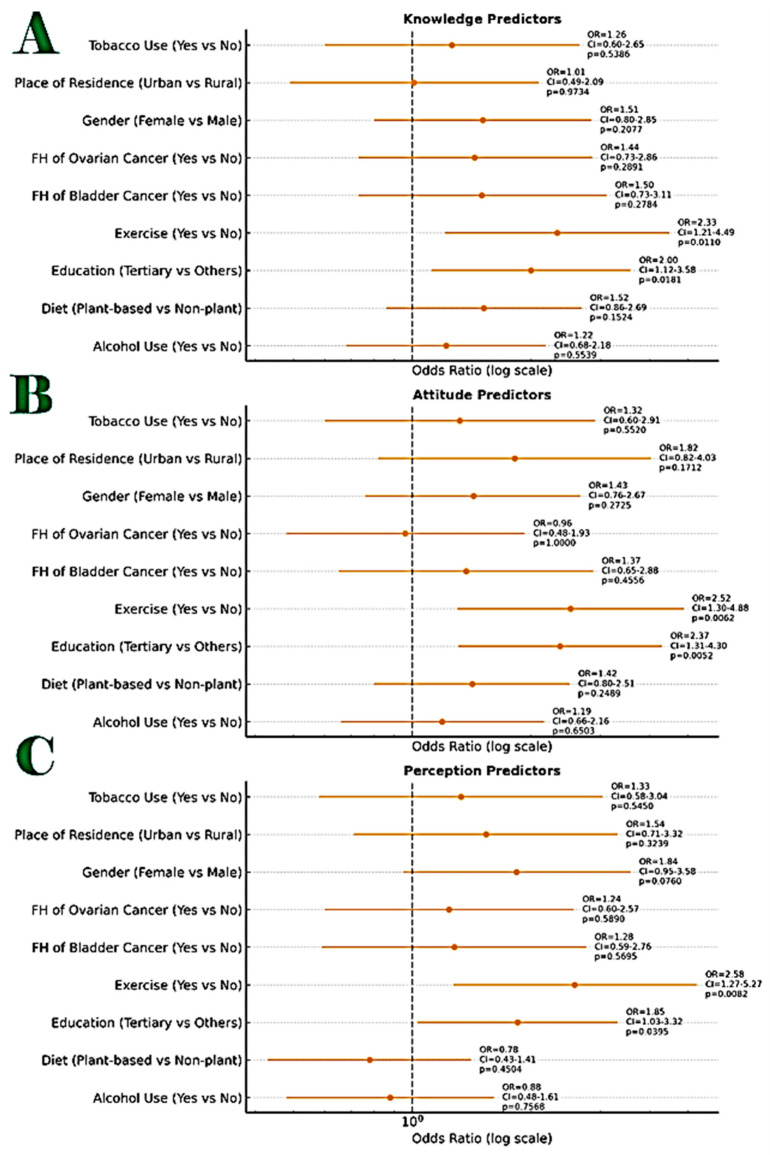
Combined annotated Forest Plot showing the odds ratios (ORs) with 95% confidence intervals (CIs) and *p*-values for predictors of knowledge, attitudes, and perceptions (KAPS) of Study participants as it relates to their sociodemographic and lifestyle characteristics. Figure Legend: (**A**). Good Knowledge (**top panel**), (**B**). Positive Attitude (**middle panel**), (**C**). Positive Perception (**bottom panel**). Each horizontal line represents a 95% confidence interval around the OR for a given predictor. The vertical dashed line at OR = 1 indicates no association. Markers to the right of the line (OR > 1) indicate increased odds of the outcome for the reference group. Annotated labels include: OR value, Confidence interval range, Exact *p*-value. The Statistically significant predictors (*p* < 0.05) were Knowledge: Tertiary education, Exercise, Attitude: Tertiary education, Perception: Tertiary education, Exercise. The odds ratios were calculated from 2 × 2 contingency tables using Fisher’s Exact Test and are displayed on a logarithmic scale for interpretability. In the Knowledge domain, two factors showed statistically significant positive associations: Tertiary education (OR = 2.37; 95% CI: 1.29–4.35; *p* = 0.0052) and Regular exercise (OR = 2.14; 95% CI: 1.11–4.14; *p* = 0.0225). In the Attitude domain, only tertiary education remained statistically significant (OR = 2.37; 95% CI: 1.29–4.35; *p* = 0.0052). In the Perception domain, both tertiary education and exercise were again found to be significantly associated with correct perception (OR = 1.85; 95% CI: 1.03–3.32; *p* = 0.0395), Regular exercise (OR = 2.57; 95% CI: 1.22–5.41; *p* = 0.0132). Other variables-such as gender, place of residence, dietary pattern, tobacco use, alcohol use, and family history of cancer-showed non-significant associations across the three domains. FH, denotes, family history.

The details of the model outputs for all the demographic variables are provided in Table 3 and visually summarized in Figure 3.

Comparative Effectiveness of S-S-LIBOG vs. HT:

Post-Intervention Changes in KAP showed that both interventions significantly improved knowledge scores, and narrowed disparities (please refer to Tables S3–S10, and Figures S3–S8 (and the accompanying write-ups) in the Supplementary Material document for the full data evidence on this).

In this main manuscript however, we detail that LIBOG yielded good knowledge increased from 35.0% to 60.0% (z = 2.85, *p* = 0.004). Health Talk (HT) also yielded good knowledge rose from 35.0% to 62.3% (z = 3.10, *p* = 0.002).

Attitudes improved markedly with LIBOG (positive attitudes rose by +23.5%), compared to smaller gains with HT (+6.8%). Perceptions improved significantly in the LIBOG group (positive perceptions +21.6%) but showed smaller, non-significant improvement in the HT group.

**Figure 4:**
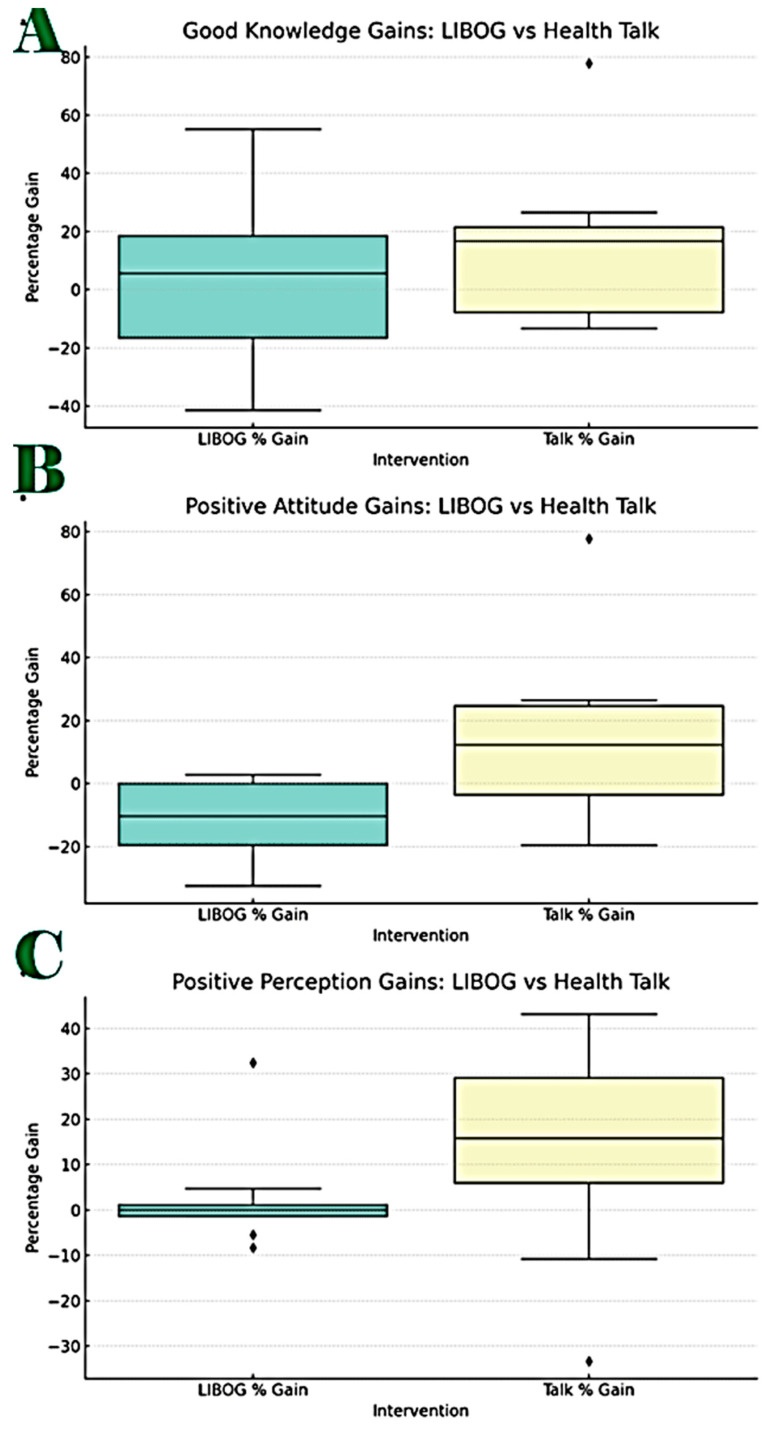
Box Plots for the subgroup ((**A**) Knowledge, (**B**) Attitudes and (**C**) Perception) Wilcoxon signed-rank test analysis: LIBOG vs. HT. Figure Legend: Good Knowledge: Number of Paired Comparisons: 9, Wilcoxon Signed-Rank Statistic (W): 18.0, *p*-value: 0.6523. Result: No statistically significant difference (Figure 4). Positive Attitude: Number of Paired Comparisons: 8, Wilcoxon signed-rank statistic (W): 5.0, *p*-value: 0.0781. Result: No statistically significant difference. Positive Perception: Number of Paired Comparisons: 8, Wilcoxon signed-rank statistic (W): 12.0, *p*-value: 0.4609. Result: No statistically significant difference.

Between-group comparison revealed no statistically significant difference in median gains (Wilcoxon W = 102.0, *p* = 0.107), suggesting overall comparable effectiveness. However, subgroup analysis (Table 4) indicated that LIBOG was more effective at narrowing gender, education, and residence-related disparities, while HT produced slightly higher knowledge gains overall. These findings are detailed in Table 4 and Table 5, and Figure 4).

Evaluation of the three hypotheses we started with (please refer to Tables S3–S10, and Figures S7 and S8 in the Supplementary Material document, for the full global comparative analysis): The study’s null hypotheses were:

**Hypothesis** **1.** *There is no difference in the proportion of participants with good knowledge of prostate cancer before and after a standard health talk intervention. We reject this hypothesis, as baseline versus post—HT differences between mean-proportions of those with a ‘good knowledge level’ had a test statistic of (z = 3.10, p = 0.002). This suggests that there was an improvement that was statistically significant (and indeed this statistically significant difference was observed in a total of 16 out of the 24 measures of central tendency and dispersion for baseline versus post-HT metrics).*

**Hypothesis** **2.** *There is no difference in the proportion of participants with good knowledge of prostate cancer before and after the session of interaction with the LIBOG game. We reject this hypothesis, as baseline versus post—LIBOG differences between mean-proportions of those with a ‘good knowledge level’ had a test statistic of (z = 2.85, p = 0.004). This suggests that there was an improvement that was statistically significant (and indeed this statistically significant difference was observed in a total of 14 out of the 24 measures of central tendency and dispersion for baseline versus post-LIBOG metrics).*

**Hypothesis** **3.** *There is no difference in the median percentage gains between LIBOG and Health Talk interventional groups in the study. We fail to reject this hypothesis, as the Test Statistic across all the KAP domains (over all the 14 sociodemographic and lifestyle characteristics), the Wilcoxon Signed-Rank Statistic (W) was 102.0, p-value = 0.107. It means that the two interventions are comparable in effectiveness. LIBOG compares favorably with HT. Since it actually offers us at least 71% of the grand gains attributable to HT in this study, it is not an inferior modality for health literacy.*

**Figure 5:**
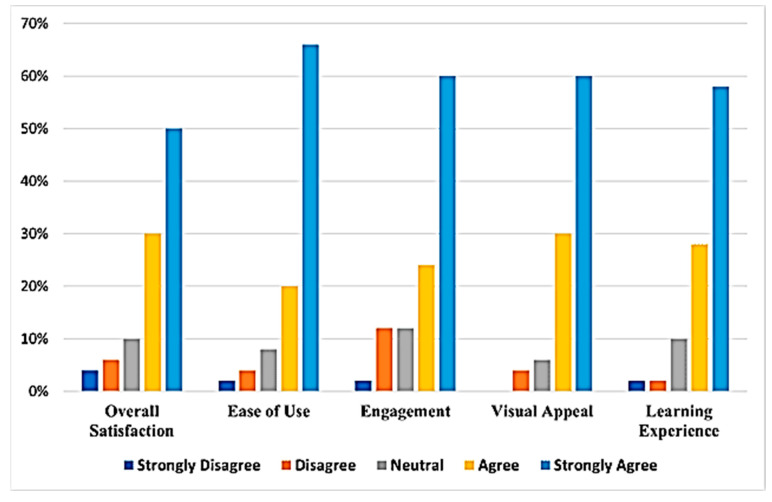
A bar chart showing participants’ usability testing results for the LIBOG game.

Literacy Game Usability Testing (Acceptability):

The usability testing also showed that at least a total of 80% of all users of the LIBOG game agreed or strongly agreed that it was easy to use, engaging, had a good visual appeal, and gave them good satisfaction and learning experience (Figure 5).

**Figure 6:**
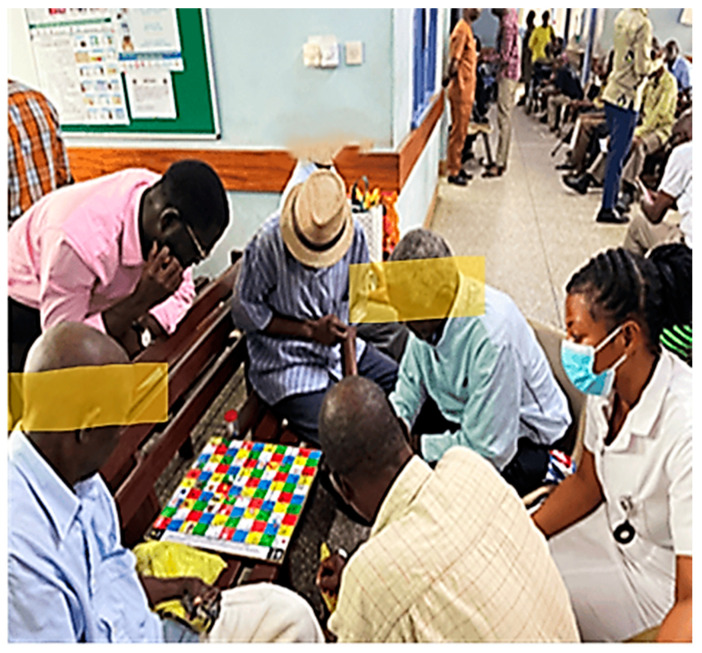
On-field education sessions using the S-S-LIBOG PCa board game and health talk. Legend: LIBOG = Literacy Board Game; PCa = Prostate Cancer.

The on-field implementation of the S-S-LIBOG is illustrated in Figure 6.

## 4. Discussion

### 4.1. Brief Summary of Results

This study evaluated the comparative effectiveness of a culturally adapted literacy board game (S-S-LIBOG) and a conventional health talk in improving prostate cancer knowledge, attitudes, and perceptions in a semi-urban Ghanaian cohort. Both approaches demonstrated significant improvements in knowledge, consistent with the long-standing role of health education in raising awareness. However, the interventions exhibited distinct profiles: while health talks achieved greater absolute gains in knowledge, the board game yielded more substantial improvements in attitudes and perceptions, which are critical precursors of behavior change. These findings highlight the value of multimodal strategies for health education, where conventional and innovative methods may complement each other rather than function as substitutes.

### 4.2. Alignment with Existing Evidence

The improvements in knowledge observed in both arms align with previous Ghanaian and regional studies showing that structured health talks, posters, and community campaigns can raise awareness of prostate cancer risk factors and symptoms [13,14,15,16,17,18,19,20]. However, the impact of these traditional methods on long-term behavior change, such as screening uptake, has been limited, often due to didactic delivery and low participant engagement. In our study, the health talk arm performed well in knowledge transfer, but less effectively in shaping attitudes and perceptions, confirming these earlier findings.

The superior performance of LIBOG in modifying attitudes and perceptions mirrors outcomes reported in other gamified interventions across Africa. Board games have been shown to enhance engagement and retention in HIV/AIDS prevention programs malaria literacy campaigns, and adolescent reproductive health education and educating emergency physicians on urogenital diseases [18,19,20,21,22,23,24,25,26,27]. The social and interactive elements of games foster peer-to-peer reinforcement, experiential learning, and emotional connection, all of which are key drivers of attitude and perception change. The present study extends this evidence to prostate cancer, an area where innovative educational strategies remain scarce.

Notably, LIBOG reduced disparities across gender, education, and residence. At baseline, males and highly educated participants demonstrated better knowledge and more favorable perceptions, reflecting documented inequities in access to health information [14,15,16,17,18,19,20,21]. Post-intervention, these gaps narrowed in the board game arm, suggesting that participatory methods may be particularly effective for engaging groups that are often left behind by conventional health talks. This supports findings from literacy interventions in low-resource contexts, which emphasize that interactive formats are better suited for heterogeneous and semi-literate populations [18,22,23,24,25,26,27].

### 4.3. Practical Implications for Ghana

The practical significance of these findings is twofold. First, LIBOG demonstrates that culturally grounded, low-cost, and community-friendly educational tools can be successfully deployed in Ghana. Unlike lectures that rely on one-way communication, the board game encourages active participation, discussion, and collective reflection, consistent with the tenets of the socioecological model/social cognitive theory [26,27,28,29]. Such engagement is vital for overcoming stigma, misconceptions, and fatalistic beliefs around prostate cancer that have been documented in Ghanaian men [13,15,16,17,18,19,20].

Second, the intervention’s high acceptability—with over 80% of participants reporting enjoyment and preference for the board game—underscores its feasibility for real-world adoption. The game’s design is adaptable: it can be translated into local languages, modified for smaller community groups, and potentially digitized for mobile platforms. In rural areas where literacy barriers are higher, the interactive format may ensure inclusivity by allowing oral facilitation. Thus, LIBOG could serve as an adjunct to conventional talks in community health programs, outreach clinics, and health-promoting schools.

For clinicians and policy-makers, the implication is clear: interactive methods should not be dismissed as mere “add-ons” but integrated deliberately into public health education. By shaping attitudes and perceptions, LIBOG could influence screening intentions, health-seeking behavior, and eventually stage at diagnosis—outcomes of critical importance in Ghana, where late presentation remains the norm.

### 4.4. Contributions to the Field

Beyond the local context, this study contributes to the growing literature on gamification in health promotion. It provides one of the first empirical evaluations of a theory-driven, culturally adapted board game for prostate cancer education in Africa. By grounding the intervention in SCT and SEM [26,27,28,29], the study illustrates how behavioral theory can be operationalized in simple community tools. The findings also highlight that effectiveness is multidimensional: while knowledge gains are important, shifting attitudes and perceptions may be equally or more valuable for driving long-term change. This balanced view advances the discourse on how effectiveness should be measured in health literacy interventions.

The 17 Other LIBOGs: In addition to the prostate cancer LIBOG, seventeen other S-S-LIBOGs have been developed, targeting a wide range of public health concerns. These include adolescent sexual and reproductive health, asthma, antimicrobial resistance, breast cancer, breastfeeding and the Baby-Friendly Hospital Initiative, cervical cancer, diabetes mellitus, hypertension, family planning, hematuria and bladder cancer, kidney diseases, male circumcision complications, child malnutrition, mental health, menstrual health, prostate cancer, and sickle cell disease. There is also the digital version of the PCa LIBOG. Each of these was modeled after the original LIBOG designed for prostate cancer [22] and adapted for the respective disease focus. They are all underpinned by the theoretical foundations of Social Cognitive Theory (SCT) [27] and the Socio-Ecological Model (SEM) [28].

In this paper, these LIBOGs are being introduced for the first time. Links to access all the games and their accompanying manuals (with the game rules and educational questions) are provided in the Supplementary Materials document (Section S14). While the authors are currently evaluating each LIBOG in a stepwise manner, all researchers are welcome and encouraged to download, adapt, and test these tools in their own settings and among their target populations. Findings from such adaptations can be reported and published independently. The entire LIBOG suite is intended as an open-access resource for shared learning, collaboration, and improved public health outcomes

### 4.5. Limitations, Biases, and Applications

Several limitations should be noted. First, the attrition rate (28.4%) may have introduced selection bias and reduced statistical power, particularly in subgroup analyses. Second, the sample was drawn from a semi-urban population with disproportionately high tertiary education representation, which limits generalizability to rural or less-educated groups. Third, the short follow-up period did not allow for evaluation of long-term retention or translation into actual screening behavior, which remains the ultimate goal of prostate cancer education. In addition, although baseline and post-intervention cohorts were matched as closely as possible, full randomization and blinding were not feasible in this community-based setting, raising the possibility of allocation bias. Finally, reliance on self-reported measures introduces risk of social desirability bias, especially in attitudes and perceptions.

An additional limitation of this study is that the trial registration was performed retrospectively (PACTR202510512711680). The delay arose from administrative processes between ethics approval and formal registry acceptance. Nonetheless, the study adhered strictly to a pre-specified protocol and analysis plan developed prior to data collection, and no outcome measures or endpoints were modified thereafter. Consequently, the retrospective nature of the registration is not expected to have introduced systematic bias or affected the integrity of the findings.

Despite these limitations, the study has important applications. For research, it underscores the need for larger and more diverse samples, longer follow-up, and inclusion of objective outcomes such as prostate-specific antigen (PSA) testing rates. For clinical practice, it demonstrates that board games are not inferior to health talks and can add unique value by addressing psychosocial dimensions of cancer literacy. For policy, it suggests that ministries of health and cancer control programs could integrate gamified approaches into national education campaigns, particularly in communities where stigma and fatalism remain strong barriers to early detection.

## 5. Conclusions

This study demonstrated that both the literacy board game (S-S-LIBOG) and conventional health talk are effective tools for improving prostate cancer awareness in Ghana. While health talks produced greater knowledge gains, LIBOG was more effective in shaping positive attitudes and perceptions, dimensions that are critical for promoting screening intentions and behavior change. The interactive, culturally adapted nature of LIBOG makes it a feasible and scalable adjunct to conventional community education methods.

We recommend that prostate cancer control programs in Ghana and similar settings integrate board-game-based education into community outreach, health-promoting schools, and men’s health initiatives. Future research should evaluate long-term outcomes, including knowledge retention and actual screening uptake, and test the effectiveness of LIBOG in rural and low-literacy populations. By complementing traditional health talks with interactive approaches such as LIBOG, prostate cancer education can become more inclusive, engaging, and impactful, ultimately contributing to earlier detection and reduced mortality.

## Figures and Tables

**Table 1:** Baseline sociodemographic and lifestyle characteristics of study participants.

Variable	Category	LIBOG (n = 80)	Health Talk (n = 61)	Total (n = 141)
Age group	≤30 years	28 (35.0%)	20 (32.8%)	48 (34.0%)
	31–50 years	40 (50.0%)	26 (42.6%)	66 (46.8%)
	≥51 years	12 (15.0%)	15 (24.6%)	27 (19.1%)
Gender	Male	54 (67.5%)	37 (60.7%)	91 (64.5%)
	Female	26 (32.5%)	24 (39.3%)	50 (35.5%)
Education	Tertiary	66 (82.5%)	49 (80.3%)	115 (81.7%)
	Secondary/Primary	14 (17.5%)	12 (19.7%)	26 (18.3%)
Residence	Urban	38 (47.5%)	27 (44.3%)	65 (46.2%)
	Rural	22 (27.5%)	20 (32.8%)	42 (29.9%)
	Suburban	20 (25.0%)	14 (23.0%)	34 (23.9%)

Family history of PCa was present in 4.6%, while 8.6% reported BRCa, 2.5% OVCa, 4.6% BLCa, and 3.0% gastrointestinal can GIT Ca (in relatives).

**Table 2:** Baseline knowledge, attitudes, and perceptions (KAP) by sociodemographic and lifestyle factors.

Variable	Subgroup	Good Knowledge %	Poor Knowledge %	Good Attitude %	Poor Attitude %	Good Perception %	Poor Perception %
Age	Young	49.32	45.21	60.27	23.29	42.47	46.58
Age	Middle	54.78	39.13	50.43	32.17	39.13	42.61
Age	Old	33.33	55.56	55.56	44.44	44.44	55.56
Gender	Female	62.07	34.48	51.72	25.86	48.28	43.1
Gender	Male	51.97	40.16	60.63	24.41	40.94	48.82
Gender	Other	0	100	0	100	0	8.33
Education	Primary	16.67	66.67	33.33	66.67	33.33	66.67
Education	Secondary	21.05	73.68	26.32	73.68	5.26	31.58
Education	Tertiary	55.9	38.51	58.39	22.36	45.96	44.1
Education	Postgraduate	63.64	27.27	54.55	36.36	27.27	63.64
Ethnicity	Akan	58.49	35.85	64.15	18.87	41.51	49.06
Ethnicity	Ga	61.54	38.46	38.46	23.08	38.46	53.85
Ethnicity	Ewe	47.56	45.12	57.32	30.49	43.9	46.34
Ethnicity	Northern Ghanaian	59.26	33.33	62.96	18.52	44.44	40.74
Ethnicity	Guan	66.67	33.33	66.67	0	100	0
Ethnicity	Other	31.58	63.16	10.53	78.95	10.53	31.58
Residence	Urban	65.93	30.77	60.44	20.88	43.96	42.86
Residence	Suburban	38.3	51.06	42.55	51.06	31.91	40.43
Residence	Rural	40.68	52.54	54.24	25.42	42.37	50.85
Tobacco	Never	54.64	38.8	57.92	24.59	42.62	47.54
Tobacco	Former User	15.38	84.62	7.69	92.31	7.69	7.69
Tobacco	Current User	0	100	0	100	100	0
Alcohol	Never	56.93	37.23	56.93	25.55	40.15	48.91
Alcohol	Former User	51.06	40.43	59.57	23.4	51.06	42.55
Alcohol	Current User	0	100	7.69	92.31	7.69	7.69
Family History PCa	Yes	44.44	44.44	44.44	44.44	33.33	22.22
Family History PCa	No	52.13	42.02	54.79	28.72	40.96	45.74
Family History BRCa	Yes	35.29	58.82	35.29	41.18	35.29	29.41
Family History BRCa	No	53.33	40.56	56.11	28.33	41.11	46.11
Family History OVCa	Yes	0	100	0	100	0	0
Family History OVCa	No	53.13	40.63	55.73	27.6	41.67	45.83
Family History BLCa	Yes	11.11	88.89	0	100	0	22.22
Family History BLCa	No	53.72	39.89	56.91	26.06	42.55	45.74
Family History GIT Ca	Yes	16.67	83.33	16.67	66.67	16.67	33.33
Family History GIT Ca	No	52.63	41.05	55.26	28.42	41.58	44.74
Exercise	Sedentary	43.86	47.37	56.14	33.33	36.84	49.12
Exercise	Light	60	32.5	61.25	18.75	45	45
Exercise	Moderate	61.54	35.9	48.72	28.21	46.15	51.28
Exercise	Active	23.81	76.19	33.33	61.9	23.81	19.05
Diet	Fatty	66.67	33.33	66.67	0	0	100
Diet	Vegetarian	18.75	81.25	25	75	25	6.25
Diet	Mixed	54.49	38.76	56.74	25.84	42.7	47.19

Legend: PCa = Prostate Cancer; BRCa = Breast Cancer; OVCa = Ovarian Cancer; BLCa = Bladder Cancer; GIT Ca = Gastrointestinal Tract Cancer. Note that percentages for moderate attributes are not presented in the table.

**Table 3:** Multivariate logistic regression of predictors of knowledge, attitudes, and perceptions.

Outcome	Predictor	AOR	95% CI	*p*-Value
Knowledge	Exercise (regular vs. none)	1.45	1.06–1.98	0.021
Attitudes	Male gender	2.56	1.30–5.01	0.006
	Higher income	1.34	1.01–1.77	0.040
	Exercise (regular vs. none)	1.58	1.15–2.17	0.005
Perceptions	Male gender	16.84	4.92–57.63	<0.001

Legend: AOR = Adjusted Odds Ratio; CI = Confidence Interval.

**Table 4:** Pre- and post-intervention changes in knowledge, attitudes, and perceptions within and between arms.

Outcome	Group	Pre (%)	Post (%)	*p*-Value
Knowledge	LIBOG	35.0	60.0	0.004
	Health Talk	35.0	62.3	0.002
Attitudes	LIBOG	51.2	74.7	0.006
	Health Talk	56.8	63.6	0.145 (NS)
Perceptions	LIBOG	40.3	61.9	<0.001
	Health Talk	42.1	48.9	0.118

Legend: LIBOG = Literacy Board Game; *p*-values derived from two-proportion Z-tests; *p* = *p*-value; NS = not statistically significant.

**Table 5:** The sub-domain statistics were as shown for the Wilcoxon signed-rank test.

KAP Domain	Wilcoxon W	*p*-Value	n (Pairs)	Conclusion
Good Knowledge	18.0	0.6523	9	Not significant
Positive Attitude	5.0	0.0781	8	Not significant
Positive Perception	12.0	0.4609	8	Not significant

Legend: W = Wilcoxon signed-rank test.

## Data Availability

The datasets generated and analyzed during the current study are not publicly available due to participant confidentiality safeguards, but anonymized data may be made available from the corresponding author on reasonable request and with permission from the Ho Teaching Hospital Research Ethics Committee.

## Supplementary Materials

The following supporting information can be downloaded at: https://www.mdpi.com/article/10.3390/healthcare13233135/s1, Figure S1. The Socio-Ecological Model of Health. Figure S2. The Social Cognitive Theory. Table S1. Multivariate logistic regression results. Table S2. Variable coding for the models. Table S3. Percentage gains post-interventions (LIBOG and talk). Figure S3. Comparative performance of the LIBOG game and health talk interventions on knowledge, attitude, and perception scores. Figure S4. Post-LIBOG intervention forest plots for KAP odds ratios. Figure S5. Percentage changes in good knowledge, positive attitude, and positive perceptions after interventions with LIBOG or health talk (HT), disaggregated by gender. Figure S6. Post health talk intervention forest plots for KAP odds ratios. Figure S7. Bar chart showing comparative gains in knowledge, attitude, and perception (altogether)—LIBOG vs. health talk and forest plot. Statistical significance of improvement differences between LIBOG and talk. Table S4. Comparatives for the measures of central tendencies (mean, median, mode) and measures of dispersion (range and standard deviation) for all KAP baseline values and KAP changes across all the 14 sociodemographic and lifestyle factors studied after LIBOG and HT interventions. Table S5. Knowledge domain: baseline, post-LIBOG and post-HT; mean, median, mode, range, standard deviation, and statistically significant differences. Table S6. Attitude domain: baseline, post-LIBOG and post-HT; mean, median, mode, range, standard deviation and statistically significant differences. Table S7. Perception domain: baseline, post-LIBOG and post-HT; mean, median, mode, range, standard deviation and statistically significant differences. Table S8. Table for intervention by health talk; aggregation of percentage improvements across 5 measures of central tendency and dispersion (mean, median, mode, range, standard deviation), across all three KAPS over the 14 socio-demographic parameters studied. Table S9. Table for intervention by LIBOG aggregation of percentage improvements across 5 measures of central tendency and dispersion (mean, median, mode, range, standard deviation), across all three KAPS, over the 14 socio-demographic parameters studied. Table S10. Comparative insights for LIBOG vs. HT. Figure S8. Box plots for the Wilcoxon Signed Rank Test analysis: LIBOG vs. HT. Refs. [27,28,33] are cited in the supplementary materials.

## Appendix A

**Table A1:** Study questionnaires. PUSQ: Source: adapted and modified from uiuxtrend.com.

Demography and Biodata
Age (years)
Gender (Male/Female)
Ethnicity
Height (cm)
Weight (kg)
Body Mass Index (BMI) (auto-calculated)
Residence (Urban/Suburban/Rural)
Tobacco Use (Never/Former/Current)
Alcohol Use (Never/Occasionally/Regularly)
Family History of Prostate Cancer (Yes/No)
Family History of Breast Cancer (Yes/No)
Family History of Ovarian Cancer (Yes/No)
Family History of Bladder Cancer (Yes/No)
Family History of GIT Cancer (Yes/No)
Exercise Habits (Sedentary/Light/Moderate/Active)
Diet Type (Fatty/Vegetarian/Mixed)
Educational Level (Primary/Secondary/Tertiary/Postgraduate)
Occupation
Residence Description
Income Level (Low/Medium/High/Not Applicable)
KNOWLEDGE & ATTITUDE QUESTIONS (Likert 1–5)
Prostate cancer is a common health issue among men.
Regular screening can help in the early detection of prostate cancer.
Prostate-Specific Antigen (PSA) is a blood test used to screen for prostate cancer.
Individuals with a relative diagnosed as prostate cancer have a higher risk of contracting prostate cancer
Most prostate cancer patients may show no signs/symptoms early, so screening is advisable
Most prostate cancer patients would show clear signs/symptoms early, so screening is not advisable
Some men with prostate cancer may have urinary challenges
Prostate cancer affects young men rather than elderly
Prostate cancer affects mostly men 50 years and above
Prostate cancer is more common in Africans and African Americans than Whites
Prostate cancer is more common amongst Whites than Blacks
Frequently recurring low back pain might indicate prostate cancer
Older people over 80 years don’t need prostate cancer screening tests
Some prostate cancer treatments complicate urinary continence in men
Some prostate cancer treatments affect sexual abilities in men
Some prostate cancer treatments restrict driving ability
Some men might die from prostate cancer, while others would not
An abnormal PSA test result absolutely indicates prostate cancer
Prostate cancer might be detected despite normal PSA
Prostate cancer might progress slowly in some men
Prostate cancer is best treated when diagnosed early
There is hope for prevention, control and treatment of prostate cancer if detected early
PSSUQ: Post-Study System Usability Questionnaire (Likert 1–5)
The game was easy to play.
I felt comfortable navigating the game controls.
I could complete my objectives quickly in the game.
The game was engaging and kept my interest.
I am satisfied with my overall experience in the game.
I would recommend this game to others.
The game’s tutorials or instructions were helpful.
I didn’t need help to progress in the game.
The game provided clear feedback on my performance.
I found the game’s storyline engaging and clear.
The graphics and visuals enhanced my gaming experience.
I felt the game mechanics were intuitive.
The game met my expectations for entertainment.
The game was educative.
Overall, I have a positive impression of the game.
I would play this game again in the future.
Suggestions for Improvement (open-ended)
