# Supplementary material for: Evaluating a Behavioural Theory-Based Board Game (S-S-LIBOG) Against Traditional Health Talk (HT) in Prostate Cancer Education: Findings from a Quasi-Experimental Study, Plus Introducing 17 Other S-S-LIBOGs
Source: Healthcare (Basel). 2025 Dec 2;13(23):3135. doi: 10.3390/healthcare13233135 (PMC12692412; doi:10.3390/healthcare13233135)
Supplement: Supplementary file 1 [file healthcare-13-03135-s001.zip › healthcare-3843868-supplementary.pdf]

# Supplementary Material

*. S1.0 Conceptual Framework*

Narrative on the Conceptual Framework Diagrams:

In this study, the Socio-Ecological Model (SEM) serves as a foundational framework for understanding the multifaceted influences on prostate cancer awareness and education. The SEM posits that health behaviors are shaped by a complex interplay of individual, interpersonal, community, and societal factors (McLeroy et al., 1988 [27, 28]).

At the individual level, personal knowledge and attitudes toward prostate cancer significantly influence health-seeking behaviors. Interpersonal factors, such as family and peer support, are crucial in shaping beliefs and encouraging proactive health measures [27, 28]. The community level includes local resources, health services, and cultural norms that either facilitate or hinder access to prostate cancer education.

Moreover, the broader societal context—including policies, legislation, and media representations—can greatly impact prostate cancer awareness initiatives [27, 28]. By integrating SEM with Social Cognitive Theory (SCT), this study highlights the importance of observational learning (vicarious learning, operant conditioning (rewards and punishments creating positive and negative reinforcements)), environmental influences and self-efficacy, particularly within the educational board game intervention. This dual framework emphasizes the necessity of addressing both personal and contextual factors in enhancing educational outcomes and improving health behaviors related to prostate cancer in South-Eastern Ghana [18-30].

This comprehensive approach not only facilitates a deeper understanding of the barriers to awareness but also informs the design of targeted interventions that leverage social dynamics and community engagement [27, 28]. Thus, the SEM-SCT framework could be pivotal in evaluating the effectiveness of innovative educational strategies compared to traditional methods (see Figs. S1 and S2).

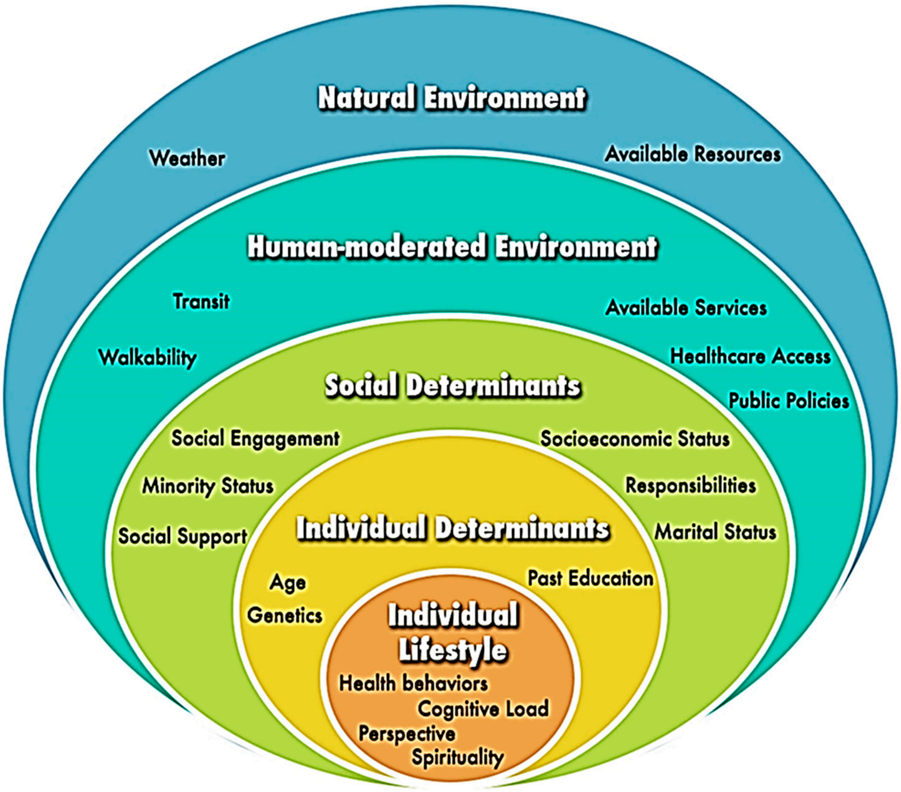


**Figure S1.** The Socio-Ecological Model of Health (Adapted from McLeroy et al, 1988). Source: McLeroy, K. R., Bibeau, D., Steckler, A., & Glanz, K. (1988). An ecological perspective on health promotion programs. Health Education Quarterly, 15(4), pages 351–377 [28].

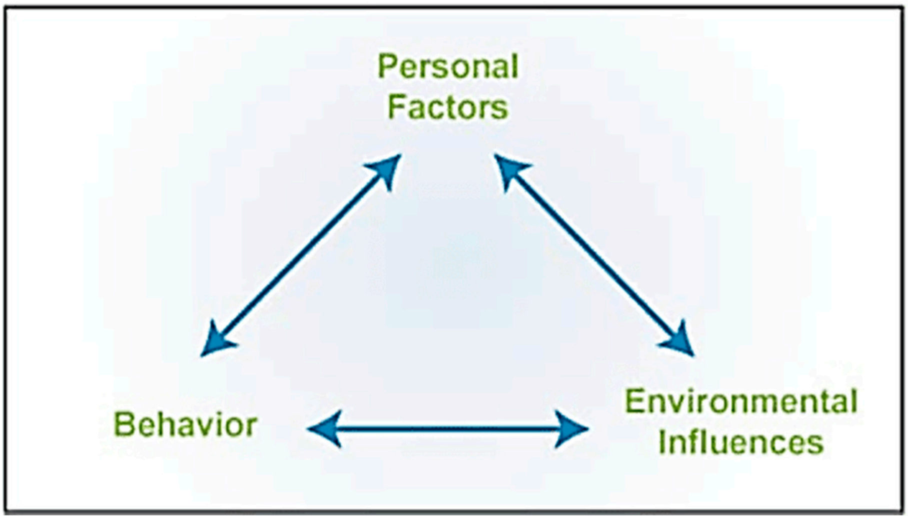


**Figure S2.** The Social Cognitive Theory. Source: Bandura, A., (1986). Social Foundations of Thought and Action: A Social Cognitive Theory. Prentice-Hall. P. 24. Key constructs of social cognitive theory that are relevant to

health behavior change interventions include observational learning, reinforcement, self-control, and self-efficacy.

S1.1 Why two conceptual frameworks? In this study, using two conceptual frameworks—the Socio-Ecological Model (SEM) and Social Cognitive Theory (SCT)—is both justified and essential given the complexity of the educational intervention. SEM addresses the multi-level environmental factors (individual, interpersonal, community, societal) influencing prostate cancer awareness, while SCT focuses on individual learning processes, behavior change, and self-efficacy. At the same time, keeping them separate, and not fused, allows for a clear distinction between the broader contextual influences (SEM) and individual cognitive mechanisms (SCT), which both contribute uniquely to understanding the effectiveness of the board game.

The approach also aligns with 'Intervention Mapping's social-ecological approach', which emphasizes the interplay between individual behaviors and environmental factors [29,30]. By integrating SEM and SCT, we gain a comprehensive understanding of both individual and contextual factors influencing health behavior change, ensuring a robust analysis of the board game's impact in South-Eastern Ghana.

*S2.0 Study Design and Participants*

Study Design: The study was a Single-Camp Quasi-Experimental Interventional Study and a two-arm comparative effectiveness study employing pre-intervention and post-intervention tests to measure and compare the specific impacts of two different methods of public education on Prostate cancer. The two arms involved were the study arm and a control arm.

Study arm: The study arm involved the use of the prostate cancer snakes, ladder, and arrows ludo game as an educational tool (pictured in Figure 2 of the main manuscript, and the link to its accompanying manuals is also below): https://drive.google.com/drive/folders/1KMSfB9k4JNNjox9f92TC6Kaj09daVYdV?usp=sharing

The LIBOG uses an interactive and relaxed interface to create health literacy. It is a journey of life from step 1 to step 100, with points of challenge (or trial points where one could rise or fall). These points have been tied to rewards that could help keep one afloat or on course, once they are able to answer a standard simplified question on prostate cancer correctly. The questions (400) are set in the manuals with the corresponding answers. These points on the board game apply social learning concepts like positive reinforcement, negative reinforcement, operant conditioning and vicarious learning to create the learning experience. The game can be

played by one to four active players at a time, and each player may have their supporters (passive players) backing them and joining to answer questions as well. Turn-taking is allowed as well. With all these, a socially engineered setting of learning which allows the tenets and pillars of social cognitive theory and the socio-ecological model (like peer influence, family influence, community influence etc.) in modifying the individual person's health behaviors come into play in order to hopefully improve the outcomes of learning and behavioral change communication. The full manual of the game is provided at the link above. One neutral person may serve as the facilitator who moderates the game with the guidance of the LIBOG game manual. At the trial points, the question that a player may be asked is decided through random selection (the player mentions any unanswered question from 1 to 400, then the facilitator administers/reads out that question from the manual).

S2.1 Board Game (S-S-LIBOG) Design summary:

The S-S-LIBOG is an interactive, snakes and ladders ludo-style board game adapted for prostate cancer literacy. Players advance along numbered squares, encountering trial points linked to prostate cancer questions. Correct answers allow progression (reinforcement), while incorrect responses may result in setbacks. The game incorporates principles of peer learning, family/community engagement, and operant conditioning.

The board accommodates 1–4 players, with facilitators moderating questions. A total of 400 prostate cancer-related questions and answers are included in the manual. The design draws inspiration from previous malaria literacy board games (Boateng et al., 2021) and health-education game prototypes in HIV and urological education.

Full rules and question banks are available via the provided link, and upon request.

Control arm: The control arm employed a didactic prostate cancer talk (with standard PowerPoint slides available at the following link: https://drive.google.com/file/d/1AufuUQsTqzOjaLxgy-m6gjwKNtGtCEfr/view?usp=sharing) as the mode of education. Participants were randomly assigned to the various arms through the toss of a dice.

S2.2 Sample Size Determination

Sample size was determined for a two-arm comparison of proportions (good knowledge), assuming a 20-percentage-point absolute improvement from baseline, at least for the control and

intervention groups [31], a two-sided significance level of 5% ($\alpha$ = 0.05), and 80% power ($\beta$ = 0.20). The formula applied was the one for sample size calculation for comparing two independent proportions (two-sided Z-test, equal allocation by Fleiss et al., 2003) [32].

n_per group = [2 × $\bar{p}$(1 − $\bar{p}$) × (Z_(1-$\alpha$/2) + Z_(1-$\beta$))$^2$] / ($p_2$ − $p_1$)$^2$

where $\bar{p}$ = ($p_1$ + $p_2$)/2, $p_1$ and $p_2$ are the expected proportions in control and intervention groups, and Z_(1-$\alpha$/2) = 1.96, Z_(1-$\beta$) = 0.84. With baseline knowledge $p_1$ = 0.35 and anticipated post-intervention knowledge $p_2$ = 0.55, we obtained n ≈ 97 per group (total ≈ 194). A sensitivity analysis indicated that an improvement of ~21 percentage points would require ~89 per group (total ≈ 178), consistent with our reported minimum requirement.

Allowing for 10% attrition inflated the requirement to ≈ 216. Considering logistical constraints, we set a target enrollment of 200 participants. This balance maintained adequate statistical power while remaining feasible in the study context.

S3.0 RESULTS:

The baseline pretest dataset comprised a total of 197 participants categorized by various socio-demographic and behavioral characteristics, with knowledge, attitude, and perception (KAP) outcomes relating to prostate cancer awareness. These are shown in Tables *1 and 2,* in the main manuscript document.

S3.1 Following the above age categorization, where 37.1% of participants were classified as "young" (n = 73), 58.4% as "middle-aged" (n = 115), and 4.6% as "old" (n = 9), an analysis of prostate cancer knowledge levels across these age groups showed that good knowledge level was present in 49.3% of the young, 54.8% of the middle-aged, and 33.3% of the old. Poor knowledge was more pronounced among the old (55.6%) and the young (45.2%) compared to the middle-aged (39.1%). However, the association between age and knowledge level category was not statistically significant ($\chi^2$ = 2.0891, p = 0.719). In terms of attitude, the young demonstrated a higher proportion of positive attitudes (60.3%) compared to middle-aged (50.4%) and older participants (55.6%), with a non-significant association ($\chi^2$ = 4.2741, p = 0.370). Similarly, perception categories did not differ significantly by age ($\chi^2$ = 3.5696, p = 0.467), although neutral perceptions were more common in the middle-aged group (18.3%) than in the young (11.0%) and old (0%).

Knowledge scores showed significant variation across gender ($\chi^2$ = 19.9202, p = 0.001). While 62.1% of females demonstrated a good

level of knowledge, this was lower among males (52.0%) and entirely absent among those identifying as "other," all of whom exhibited poor knowledge. A slightly inversed (and still statistically significant) pattern was observed in attitude categories ($\chi^2 = 32.5956$, $p < 0.001$), with positive attitudes highest among males (60.6%) and females (51.7%), but absent among the "other" gender group, all of whom had negative attitudes. Perception scores were also significantly associated with gender ($\chi^2 = 61.2492$, $p < 0.001$), with the "other" gender group predominantly neutral (91.7%), while males and females were more evenly distributed across categories.

From the above, educational level was heavily skewed toward tertiary education (81.7%), with only small proportions having primary (3.1%), secondary (9.6%), or postgraduate (5.6%) education. A statistically significant association was observed between education level and knowledge ($\chi^2 = 13.4968$, $p = 0.036$). Good knowledge was most common among postgraduates (63.6%) and those with tertiary education (55.9%), but much lower among those with only primary (16.7%) or secondary (21.1%) education. Attitudes also varied significantly with education ($\chi^2 = 27.4030$, $p < 0.001$), with positive attitudes seen in 58.4% of tertiary-educated respondents and 54.6% of postgraduates, compared to just 25.0% and 33.3% of secondary- and primary-level participants, respectively. Perceptions followed a similar pattern ($\chi^2 = 43.7216$, $p < 0.001$), with positive perceptions more prevalent among tertiary-educated respondents (45.9%).

No statistically significant association was observed between ethnicity and prostate knowledge levels ($\chi^2 = 7.4478$, $p = 0.683$), although knowledge appeared highest among the Guan (66.7%) and Ga (61.5%). However, both attitude ($\chi^2 = 34.6644$, $p < 0.001$) and perception concerning prostate cancer ($\chi^2 = 37.1847$, $p < 0.001$) were strongly associated with ethnicity. Positive attitudes were most frequent among Guan (66.7%) and Northern Ghanaians (63.0%), while the "Other" ethnic group had a high concentration of negative attitudes (78.9%) and poor perception scores (only 10.5% with positive perception).

Urban residents showed the highest rate of good knowledge level on prostate cancer (65.9%) compared to suburban (38.3%) and rural (40.7%) participants. This association was statistically significant ($\chi^2 = 14.5797$, $p = 0.006$). Positive attitudes also followed a similar trend ($\chi^2 = 15.6341$, $p = 0.004$), being most prevalent among urban (60.4%) and rural (54.2%) residents. Perceptions differed significantly as well ($\chi^2 = 9.9496$, $p = 0.041$), with urban and rural dwellers having similar positive perception levels (43.9% and 42.4%, respectively), while suburban residents showed a more neutral outlook (27.7%).

The tobacco user grouping was significantly associated with good knowledge level on prostate cancer ($\chi^2 = 11.8984$, $p = 0.018$), positive attitude ($\chi^2 = 29.2372$, $p < 0.001$), and positive perception ($\chi^2 = 55.5389$, $p < 0.001$). Good knowledge was least common among former and current users. Among former users, 84.6% had poor knowledge, and only 7.7% exhibited a positive attitude. Notably, all current users reported negative attitudes and perceptions.

Alcohol use was reported as never by 69.5% of respondents, formerly by 23.9%, and currently by 6.6%. Significant differences in knowledge levels ($\chi^2 = 19.8448$, $p = 0.001$), attitude ($\chi^2 = 26.6195$, $p < 0.001$), and perception ($\chi^2 = 56.0029$, $p < 0.001$) were found. All current users (100%) had poor knowledge levels, with a vast majority expressing negative attitudes (92.3%) and perceptions (84.6% neutral) on prostate cancer. In contrast, good knowledge and positive attitudes were more frequent among teetotalers and former users.

Family history of prostate cancer (FMH) was present in 4.6% of participants but not significantly associated with disease knowledge levels ($\chi^2 = 0.5000$, $p = 0.779$) or attitude ($\chi^2 = 1.0431$, $p = 0.594$). However, there was a modest but statistically significant association with perception ($\chi^2 = 6.8469$, $p = 0.033$), as those with FMH were more likely to express neutral perceptions about the disease (44.4%) compared to those without.

Family history of breast cancer (FHBca) was not significantly associated with knowledge levels of prostate cancer ($\chi^2 = 2.2079$, $p = 0.332$) or attitude ($\chi^2 = 2.7175$, $p = 0.257$) but showed a significant link with perception ($\chi^2 = 6.4487$, $p = 0.040$), as neutral perceptions were more common among those with FHBca (35.3%).

A significant association was also found between family history of ovarian cancer (FHOca) and all three KAP outcomes. All respondents with such history had poor knowledge ($\chi^2 = 7.0463$, $p = 0.030$), negative attitudes ($\chi^2 = 12.2948$, $p = 0.002$), and neutral perceptions (100%) ($\chi^2 = 29.7198$, $p < 0.001$) about prostate cancer. Similarly, a history of bladder cancer (FHBlca) was significantly associated with prostate cancer knowledge levels ($\chi^2 = 8.4802$, $p = 0.014$), attitude ($\chi^2 = 22.6015$, $p < 0.001$), and perception ($\chi^2 = 30.3669$, $p < 0.001$), with very poor KAP outcomes among those with such family history.

Family history of gastrointestinal cancers (GITca) was relatively rare (3.0%) and not significantly associated with prostate knowledge levels ($\chi^2 = 4.2942$, $p = 0.117$) or attitude ($\chi^2 = 4.4776$, $p = 0.107$), though perception showed a modestly significant association ($\chi^2 = 6.2392$, $p = 0.044$).

Exercise habits were mostly light (40.6%) or sedentary (28.9%), with fewer reporting moderate (19.8%) or active (10.7%) lifestyles. Knowledge levels differed significantly with exercise levels ($\chi^2 = 16.9078$, $p = 0.010$), with good knowledge highest among those

engaging in moderate (61.5%) or light (60.0%) activity. Positive attitudes were also more prevalent among those with light (61.3%) and moderate (48.7%) activity levels ($\chi^2$ = 18.2742, p = 0.006). Perceptions varied significantly ($\chi^2$ = 36.8740, $p < 0.001$), with high neutral perceptions reported among the most active group (57.1%).

Lastly, significant associations were observed between diet type and prostate cancer knowledge levels ($\chi^2$ = 11.3794, p = 0.023), attitude ($\chi^2$ = 19.0436, p = 0.001), and perception ($\chi^2$ = 44.7034, $p < 0.001$). Poor knowledge levels and negative attitudes towards prostate cancer were most common among vegetarians, while those consuming mixed diets showed relatively higher levels of good knowledge (54.5%) and positive attitudes (56.7%). Notably, all respondents consuming only fatty foods had negative perceptions, while vegetarians were more neutral.

In summary, statistically significant associations were found between KAP outcomes and gender, education, residence, ethnicity, lifestyle factors (exercise, diet, tobacco and alcohol use), and selected family cancer histories. These baseline findings provide insight into how socio-demographic and behavioral factors influence prostate cancer awareness and dispositions and form the foundation for evaluating the subsequent interventions in this study.

Relationships between demographic parameters and knowledge, attitude, and perception

Knowledge: A forest plot was constructed to display the adjusted odds ratios (ORs), 95% confidence intervals (CIs), and p-values for the association between nine selected variables and each of the three KAP domains: knowledge, attitude, and perception toward prostate cancer risk prevention and treatment methods (Figure 3).

### *S3.2. Multivariate Analysis*

Baseline knowledge, attitude and perception levels as a function of participants' socio-demographic features were used in multivariate logistic regression analyses. Table S1 shows the outputs.

**Table S1.** Multivariate logistic regression results.

| | AOR | CI Lower | CI Upper | P-Value | Model |
|---|---|---|---|---|---|
| Intercept | 0.05 | 0.01 | 0.38 | 0.0037 | Good_Knowledge |
| Gender | 1.81 | 0.94 | 3.46 | 0.0751 | Good_Knowledge |
| Educational_Level | 0.99 | 0.54 | 1.82 | 0.9709 | Good_Knowledge |
| Income_Level | 1.14 | 0.87 | 1.5 | 0.3484 | Good_Knowledge |

| | | | | | |
|---|---|---|---|---|---|
| Exercise_Habits | 1.45 | 1.06 | 1.98 | 0.0207 | Good_Knowledge |
| Intercept | 0.03 | 0 | 0.22 | 0.0006 | Positive_Attitude |
| Gender | 2.56 | 1.3 | 5.01 | 0.0062 | Positive_Attitude |
| Educational_Level | 0.84 | 0.46 | 1.56 | 0.5864 | Positive_Attitude |
| Income_Level | 1.34 | 1.01 | 1.77 | 0.0395 | Positive_Attitude |
| Exercise_Habits | 1.58 | 1.15 | 2.17 | 0.0047 | Positive_Attitude |
| Intercept | 0 | 0 | 0.03 | 0 | Correct_Perception |
| Gender | 16.84 | 4.92 | 57.63 | 0 | Correct_Perception |
| Educational_Level | 1.28 | 0.54 | 3.02 | 0.5706 | Correct_Perception |
| Income_Level | 0.85 | 0.61 | 1.18 | 0.3179 | Correct_Perception |
| Exercise_Habits | 1.14 | 0.81 | 1.62 | 0.451 | Correct_Perception |

*S3.3. Good Knowledge*

After adjusting for socio-demographic and lifestyle variables, only exercise habits emerged as a significant predictor of good knowledge about prostate cancer. Respondents who exercised regularly had significantly higher odds of having a good knowledge level compared to those who did not (AOR = 1.45, 95% CI: 1.06-1.98, p = 0.0207). Gender showed a borderline association, with males having higher odds of good knowledge than females (AOR = 1.81, 95% CI: 0.94-3.46, p = 0.0751), although this did not reach statistical significance. Educational level and income level were not significantly associated with knowledge in this model.

*S3.4. Positive Attitude*

Significant predictors of a positive attitude toward prostate cancer screening included gender, income level, and exercise habits. Males were more than twice as likely as females to exhibit a positive attitude (AOR = 2.56, 95% CI: 1.30-5.01, p = 0.0062). Higher income levels were associated with greater odds of a positive attitude (AOR = 1.34, 95% CI: 1.01-1.77, p = 0.0395). Additionally, participants who exercised regularly were significantly more likely to have a positive attitude (AOR = 1.58, 95% CI: 1.15-2.17, p = 0.0047). Educational level was not significantly associated with the attitude towards the disease.

*S3.5. Correct Perception*

Among the predictors examined, gender was the only variable significantly associated with correct perception. Males were

substantially more likely to have correct perception scores than females (AOR = 16.84, 95% CI: 4.92-57.63, p < 0.0001). No significant associations were found between perception and educational level (AOR = 1.28, p = 0.5706), income (AOR = 0.85, p = 0.3179), or exercise habits (AOR = 1.14, p = 0.451) (Table S2).

Logistic Regression Equations in Latex Annotations (Logit Models):

Let $Y_1$ = good knowledge, $Y_2$ = positive attitude, and $Y_3$ = correct Perception; then,

\log\left(\frac{P(Y=1)}{1 - P(Y=1)}\right) = \beta_0 + \beta_1X_1 + \beta_2X_2 + \dots + \beta_nX_nlog(1−P(Y=1)P(Y=1))=β0+β1X1+β2X2+⋯+βnXn

where P(Y=1)P(Y=1)P(Y=1) is the probability of the outcome (e.g., good knowledge);

β0\beta_0β0 is the intercept;

βi\beta_iβi are the logit coefficients (ln(AOR);

XiX_iXi are the predictor variables.

Model 1: Good Knowledge (with the variable codes in Table 3),

\log\left(\frac{P(\text{Good Knowledge})}{1 - P(\text{Good Knowledge})}\right) = -2.9591 + 0.5910(\text{Gender}) - 0.0114(\text{Educational Level}) + 0.1319(\text{Income Level}) + 0.3696(\text{Exercise Habits})log(1−P(Good Knowledge)P(Good Knowledge))=−2.9591+0.5910(Gender)−0.0114(Educational Level)+0.1319(Income Level)+0.3696(Exercise Habits)

Model 2: Positive Attitude

\log\left(\frac{P(\text{Positive Attitude})}{1 - P(\text{Positive Attitude})}\right) = -3.5066 + 0.9401(\text{Gender}) - 0.1748(\text{Educational Level}) + 0.2944(\text{Income Level}) + 0.4552(\text{Exercise Habits})log(1−P(Positive Attitude)P(Positive Attitude))=−3.5066+0.9401(Gender)−0.1748(Educational Level)+0.2944(Income Level)+0.4552(Exercise Habits)

Model 3: Correct Perception

\log\left(\frac{P(\text{Correct Perception})}{1 - P(\text{Correct Perception})}\right) = -6.3479 + 2.8234(\text{Gender}) + 0.2476(\text{Educational Level}) - 0.1626(\text{Income Level}) + 0.1316(\text{Exercise Habits})log(1−P(Correct Perception)P(Correct Perception))=−6.3479+2.8234(Gender)+0.2476(Educational Level)−0.1626(Income Level)+0.1316(Exercise Habits)

**Table S2.** Variable coding for the models.

| Predictor | Coding Interpretation |
|---|---|
| Gender | 1 = Male, 2 = Female (or binary: 0 = F, 1 = M) |
| Educational Level | Ordinal (e.g., 0 = None, 1 = Primary, etc.) |
| Income Level | Ordinal (e.g., 1 = Low, 2 = Medium, 3 = High) |
| Exercise Habits | Ordinal (e.g., 0 = Never, 1 = Sometimes, etc.) |

Intervention (Post-baseline): (1) Participants' interaction with the literacy board game (study arm). (2) Participants listened to a standardized PowerPoint health talk on prostate cancer (control Arm). Assignment of participants to the respective arm was determined by systematic random sampling of the baseline participants into two groups, and the toss of a dice to determine which group experiences which intervention. After editing and validating the filled post-test questionnaires and accounting for attrition (which unfortunately was quite extreme in the post-intervention phase of the study (28.4%), the LIBOG arm had 80 participants, whilst the HT arm had 61 participants.

Post-Intervention Summary of Knowledge, Attitude, and Perception Towards Prostate Cancer: After playing/interacting with the LIBOG game intervention (Figure *6*), a comprehensive analysis was conducted across multiple demographic and behavioral variables to assess the KAP changes. The new associations/relationships between the socio-demographic/lifestyle factors and knowledge, attitude, and perception (KAP) regarding prostate cancer screening were also studied.

*S3.6. Chi-Square Analysis Post-LIBOG: Gender and Knowledge, Attitude, and Perception*

Among females, 43.14% had poor knowledge, 1.96% had moderate knowledge, and 54.9% had good knowledge. Among males, 20.69% had poor knowledge, 3.45% moderate, and 75.86% had good knowledge. Although males appeared to show a higher proportion of good knowledge, this difference remained statistically non-significant ($\chi^2(2) = 4.12$, $p = 0.127$). At baseline, there was statistical significance in this aspect. This suggested a bridge in knowledge level disparities after the intervention.

Attitude still showed a statistically significant difference ($\chi^2(2) = 9.81$, $p = 0.007$), in that 49.02% of females had a negative attitude,

19.61% neutral, and 31.37% positive attitude, whereas 16.67% of males had a negative attitude, 20.00% neutral, and 63.33% positive attitude.

Perception was no longer significantly associated with gender ($\chi^2(2) = 3.56$, $p = 0.168$), in that 45.10% of females had a negative perception, 1.96% neutral, and 52.94% a positive perception. For males, 26.67% had a negative perception, none were neutral, and 73.33% had a positive perception.

*S3.7. Education Level and Knowledge, Attitude, and Perception*

Among postgraduates, 77.78% had poor knowledge, 0.00% moderate, and 22.22% good knowledge. Among primary school participants, 25% had poor knowledge, none were moderate, and 75% had good knowledge. The highest 'good knowledge level' was seen in primary school respondents. The association became statistically non-significant ($\chi^2(6) = 8.51$, $p = 0.203$) post-intervention.

Attitudes no longer showed significant differences ($\chi^2(6) = 5.38$, $p = 0.496$). Positive attitudes were most common among postgraduates (22.22%) and secondary school respondents (25.00%). Neutral attitudes remained unchanged, while negative attitudes ranged from 25% to 66.67%.

Perception after LIBOG intervention also lacked significant association with education level ($\chi^2(6) = 8.58$, $p = 0.199$). The highest proportion of good perception (66.67%) was among tertiary and primary respondents. Neutral perceptions were very low or absent across all education levels.

*S3.8. Ethnicity and Knowledge, Attitude, and Perception*

Knowledge remained not significantly associated with ethnicity ($\chi^2(10) = 6.99$, $p = 0.726$). Good knowledge was highest among Ga (80.00%) and Guan (100.00%), and lowest among Northern Ghanaians (55.56%).

Attitude shifted and showed no statistically significant variation ($\chi^2(10) = 3.90$, $p = 0.952$). Positive attitudes were most prominent among Ga (60.00%) and Guan (100.00%), post-LIBOG.

Perception persisted in showing a significant association with ethnicity ($\chi^2(10) = 27.96$, $p = 0.002$). The highest proportion of good perception was among Guan (100%), Ga (60.00%), and Northern Ghanaians (55.56%). The lowest positive perception was among Ewes (34.15%), post-LIBOG.

Residence and knowledge, attitude, and perception associations also evened out:

Knowledge levels did not differ significantly across residence ($\chi^2(4) = 3.05$, $p = 0.549$). Rural residents had 72.73% good knowledge, suburban 38.46%, and urban 37.78%.

Attitude and perception were also not significantly associated with residence (attitude: $\chi^2(4) = 0.70$, $p = 0.952$; perception: $\chi^2(4) =$

1.72, p = 0.786). Positive attitudes and perceptions were slightly more prevalent among suburban and rural residents post-LIBOG. The LIBOG changes are shown in Table *S3* and Figure *S3*.

**Table S3.** Percentage gains post-interventions (LIBOG and talk).

| Domain | Group | KAP | LIBOG % Gain | Talk % Gain |
|---|---|---|---|---|
| Gender | Female | Good Knowledge | 11.76 | 7.32 |
| Gender | Male | Good Knowledge | 55.17 | -13.36 |
| Education | Postgrad | Good Knowledge | -41.38 | 77.78 |
| Education | Primary | Good Knowledge | 58.3 | |
| Ethnicity | Ga | Good Knowledge | 18.5 | -13.33 |
| Ethnicity | Guan | Good Knowledge | 33.3 | |
| Residence | Urban | Good Knowledge | -28.12 | 21.48 |
| Residence | Suburban | Good Knowledge | 0.16 | 19.87 |
| Residence | Rural | Good Knowledge | 32.03 | -7.73 |
| FMH | Yes | Good Knowledge | 5.6 | 16.67 |
| FMH | No | Good Knowledge | -16.57 | 26.54 |
| Gender | Female | Positive Attitude | -20.33 | 26.41 |
| Gender | Male | Positive Attitude | 2.73 | -19.58 |
| Education | Postgrad | Positive Attitude | -32.38 | 77.78 |
| Education | Primary | Positive Attitude | 66.7 | |
| Ethnicity | Guan | Positive Attitude | 33.3 | |
| Ethnicity | Northern Ghanaian | Positive Attitude | | |
| Residence | Urban | Positive Attitude | -4.84 | 0 |
| Residence | Suburban | Positive Attitude | 0 | 24.07 |
| Residence | Rural | Positive Attitude | 0 | -14.2 |
| FMH | Yes | Positive Attitude | -19.4 | 8.33 |
| FMH | No | Positive Attitude | -15.84 | 16.21 |
| Gender | Female | Positive Perception | 4.64 | 11.5 |

| | | | | |
|---|---|---|---|---|
| Gender | Male | Positive Perception | 32.43 | -10.83 |
| Education | Postgrad | Positive Perception | 0 | |
| Education | Primary | Positive Perception | 0 | -33.3 |
| Ethnicity | Guan | Positive Perception | 0 | |
| Ethnicity | Northern Ghanaian | Positive Perception | | |
| Residence | Urban | Positive Perception | 0 | 18.96 |
| Residence | Suburban | Positive Perception | 0 | 43.1 |
| Residence | Rural | Positive Perception | 0 | 12.6 |
| FMH | Yes | Positive Perception | -8.3 | 41.67 |
| FMH | No | Positive Perception | -5.44 | 24.83 |
| Education | Postgrad | Negative Attitude | -48.47 | 66.67 |
| Education | Primary | Negative Attitude | 33.3 | |
| FMH | No | Negative Attitude | -6.66 | 7.93 |
| FMH | Yes | Negative Attitude | -5.6 | 0 |
| Residence | Rural | Negative Attitude | 0 | -4.2 |
| Residence | Suburban | Negative Attitude | 0 | 40.73 |
| Residence | Urban | Negative Attitude | -3.63 | 0 |
| Education | Postgrad | Negative Perception | 0 | |
| Education | Primary | Negative Perception | -33.4 | -33.3 |
| FMH | No | Negative Perception | 11.5 | 7.89 |
| FMH | Yes | Negative Perception | 16.7 | 16.67 |
| Residence | Rural | Negative Perception | 0 | 12.6 |
| Residence | Suburban | Negative Perception | 0 | 43.1 |
| Residence | Urban | Negative Perception | 12 | 6.96 |
| Education | Postgrad | Poor Knowledge | -50.48 | 77.78 |
| Education | Primary | Poor Knowledge | 41.7 | |
| FMH | No | Poor Knowledge | -21.9 | 12.07 |

| | | | | |
|---|---|---|---|---|
| FMH | Yes | Poor Knowledge | 19.4 | -8.33 |
| Residence | Rural | Poor Knowledge | 32.03 | -7.73 |
| Residence | Suburban | Poor Knowledge | 23.24 | -3.21 |
| Residence | Urban | Poor Knowledge | -8.28 | -2.96 |

TABLE LEGEND: FMH = family history; HT = health talk; LIBOG = literacy board game.

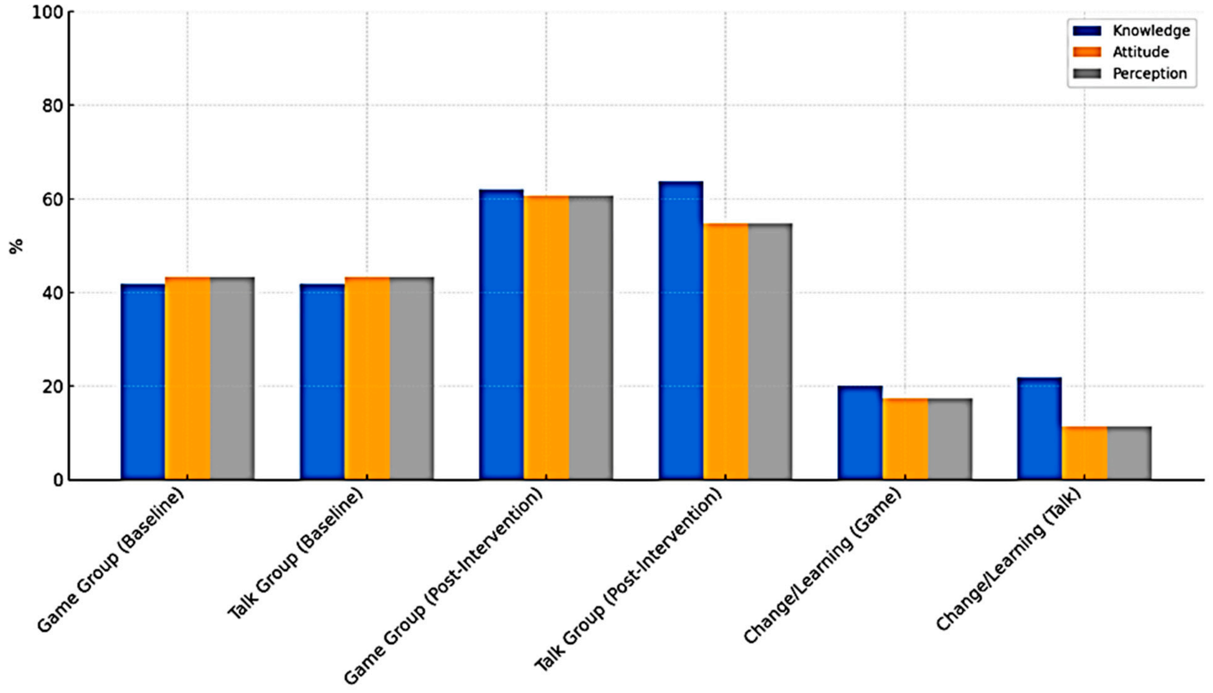


**Figure S3.** Comparative performance of the LIBOG game and health talk interventions on knowledge, attitude, and perception scores. FIGURE LEGEND: This bar chart displays the comparative performance of two educational interventions—the LIBOG game and a standard health talk—across three domains: knowledge, attitude, and perception. The chart is structured into six grouped categories: baseline scores for both the LIBOG and talk groups (shared, since all participants began from the same baseline), post-intervention scores separately for the LIBOG group and the talk group, computed improvements ("Change/Learning") in each domain for each group. Each group contains three vertically stacked bars. Blue: knowledge, orange: attitude, gray: perception. All values are expressed as percentages. The LIBOG group showed higher mean post-intervention scores in attitude and perception, and comparable gains in knowledge, while the health talk demonstrated slightly higher post-knowledge scores. The change bars (on the far right) illustrate the net gains from baseline, with the LIBOG game outperforming in affective domains (attitude and perception), indicating the

potential effectiveness of interactive educational tools in shaping psychosocial learning outcomes. Grid lines represent 10% increments to aid in visual comparison. All groupings reflect N = 80 for LIBOG, N = 61 for the talk group, and N = 197 at baseline.

*S3.9. Family History of Prostate Cancer (FMH)*

Knowledge became significantly associated with family history of prostate cancer ($\chi^2(2) = 8.75$, $p = 0.013$), in that 25% of those with family history had poor knowledge, 25% moderate, and 50% good knowledge, compared to 35.53% good knowledge among those without FMH.

Attitude was still not significantly associated (attitude: $\chi^2(2) = 1.07$, $p = 0.587$; perception, however, evened out after LIBOG: $\chi^2(2) = 0.392$, $p = 0.822$), in that 50% had a negative attitude and 25% a positive perception among those with FMH, compared to 38.96% positive perception in those without.

Family history of breast cancer (FHBca) attributes also evened out:

Knowledge ($\chi^2(2) = 3.05$, $p = 0.217$) and attitude ($\chi^2(2) = 0.46$, $p = 0.793$) were not statistically associated. However, perception was significantly associated ($\chi^2(2) = 10.42$, $p = 0.005$) with positive perception among those with FHBca at 77.78% compared to 42.25% among those without.

Family history of ovarian cancer (FHOca) attributes also evened out:

Knowledge remained highly significant ($\chi^2(2) = 39.49$, $p = 0.000$), with 100% good knowledge among the only positive case. Attitude and perception were not statistically significant, though 100% of those with FHOca had positive attitude and perception after the LIBOG intervention.

*S3.10. Family History of Bladder and Gastrointestinal (GIT) Cancer*

Also, after the intervention, both bladder and GIT family history showed highly significant associations with knowledge ($\chi^2(2) = 39.49$ and 38.99, respectively, $p < 0.001$). Good knowledge among the positive cases was at 100%. Attitudes and perceptions did not differ significantly but shifted toward complete positivity.

After the LIBOG intervention, most of the lifestyle factors evened out, narrowing disparities:

Tobacco Use: No significant associations were observed (knowledge: $\chi^2(2) = 1.88$, attitude: $\chi^2(2) = 1.33$, perception: $\chi^2(2) = 0.66$). The only current user had 100% good knowledge and positive attitude.

Alcohol use showed no significant associations. Good knowledge was highest among regular users (100%) and occasional drinkers (44.44%). Attitude showed 100% positivity in regular users.

Exercise Habits: Attitude approached significance but was not ($\chi^2(6) = 11.34$, $p = 0.078$). Positive attitude was most prominent in active exercisers (87.50%), while moderate exercisers had more negative views. Knowledge and perception showed no significant differences.

Diet Type: No significant associations were found, though higher good knowledge levels and positive attitudes were noted among vegetarians and those on fatty diets.

Regression analysis post-LIBOG: The valid odds ratios from the post-LIBOG game intervention show several statistically meaningful relationships between socio-demographic variables and the new knowledge, attitude, or perception (KAP) outcomes.

Males had significantly lower odds of demonstrating good knowledge compared to females, with an odds ratio of 0.34 (95% CI: 0.12-0.99, p = 0.047) after intervention with the LIBOG game. However, in contrast, males were significantly more likely to express a positive attitude than females, with an odds ratio of 4.16 (95% CI: 1.58-10.94, p = 0.004). Similarly, males also had significantly higher odds of having a positive perception than females, with an odds ratio of 2.79 (95% CI: 1.01-7.69, p = 0.047).

When educational background was examined, tertiary-educated participants had lower odds of good knowledge than those with only primary education (a finding that may also support a bridging of disparities by the intervention), though this difference was not statistically significant (OR = 0.53, 95% CI: 0.05-5.86, p = 0.607). Likewise, they were more likely to have a positive perception (OR = 1.89, 95% CI: 0.23-15.74, p = 0.557), though again this was not statistically significant.

In terms of residential setting, rural residents were more likely to demonstrate good knowledge compared to their urban counterparts, with an odds ratio of 4.39 (95% CI: 1.06-18.16, p = 0.041), a statistically significant finding. However, regarding attitude, rural residents were less likely to have a positive attitude than urban residents, with an odds ratio of 0.71 (95% CI: 0.17-2.89, p = 0.632), though this association was not statistically significant. Similarly, rural residents had slightly higher odds of a positive perception compared to urban dwellers, but the difference was not significant (OR = 1.23, 95% CI: 0.30-5.13, p = 0.776).

For family history of prostate cancer (FMH), participants with a positive family history had slightly higher odds of demonstrating good knowledge than those without (OR = 1.10, 95% CI: 0.16-7.34, p = 0.920), but the difference was not statistically significant. The same trend was seen for attitude (OR = 1.25, 95% CI: 0.19-8.33, p = 0.813) and perception (OR = 1.25, 95% CI: 0.19-8.33, p = 0.813), both showing no significant association between FMH and post-intervention improvements.

When examining family history of breast cancer (FHBca), participants with a positive history were more likely to demonstrate a positive perception, with an odds ratio of 4.80 (95% CI: 0.89-25.88), though the association approached but did not reach statistical significance ($p = 0.068$). This suggests a potential trend where family history of related cancers may influence perception positively after educational intervention.

Overall, the LIBOG game intervention appeared to reduce gender disparities in knowledge and significantly improve attitudes and perceptions among males. Residence and family cancer history showed varying degrees of influence, though only a few associations reached statistical significance. These findings highlight the importance of tailoring interventions to account for baseline demographic differences while demonstrating the utility of game-based education in enhancing prostate cancer screening awareness, and allied attributes (Figure S4, S5 and Table S4).

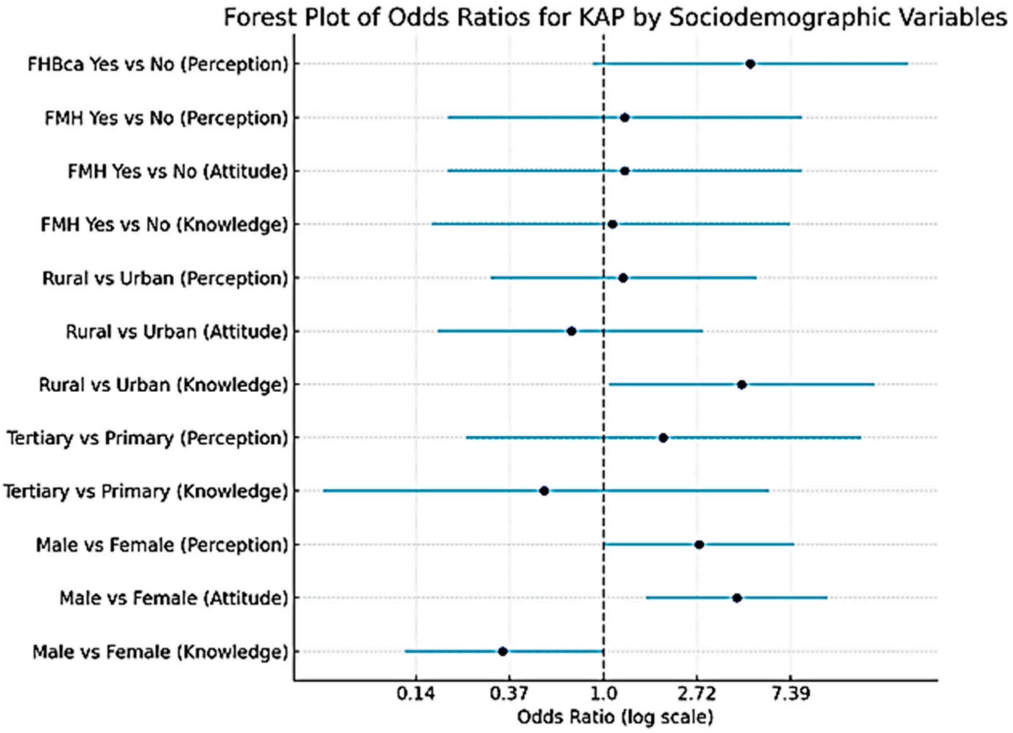


**Figure S4.** Post-LIBOG intervention forest plots for KAP odds ratios.

Knowledge, attitude and perception after listening to the PowerPoint health talk (HT) on prostate cancer (post-test results).

Chi-Square Analysis post-HT:

Gender and Knowledge, Attitude, and Perception (Post-HT intervention): After the educational talk, both female and male participants demonstrated high levels of knowledge regarding

prostate cancer. Among females, 62.22% had good knowledge, 2.22% moderate, and 35.56% poor. Similarly, 62.50% of males had good knowledge and 37.50% poor, with none reporting moderate knowledge. The distribution was nearly identical between sexes, and this was confirmed by a non-significant association ($\chi^2(2) = 0.3681$, $p = 0.832$). Table 4 depicts the post-HT percentage changes in the KAP moieties as well.

Concerning attitude, females appeared to show stronger positivity, with 57.78% having a positive attitude compared to 43.75% of males. Negative attitudes were more prevalent in males (50%) than in females (26.67%). Despite these differences, the association between gender and attitude was not statistically significant ($\chi^2(2) = 3.1687$, $p = 0.205$).

Perceptions were also favorable across both genders. Positive perception was slightly higher in females (64.44%) than in males (62.50%), and the gender difference was not statistically significant ($\chi^2(2) = 0.6066$, $p = 0.738$). Considering the fact that the pre-interventional associations were significant, it means that the intervention had bridged the pre-exiting disparities in this aspect.

*S3.11. Education and Knowledge, Attitude, and Perception Post-HT*

Participants with higher education levels exhibited generally better knowledge. Among those with postgraduate education, 100% had good knowledge. For tertiary-educated participants, 61.54% had good knowledge, and 36.54% had poor knowledge. The association between education and knowledge was not statistically significant ($\chi^2(6) = 3.2371$, $p = 0.779$). So, the talk intervention had evened out the initial disparities.

Attitudes were also largely positive across all education levels. Notably, 100% of participants with only primary education expressed a positive attitude. Tertiary-educated participants showed a balance, with 50% expressing positive attitudes, 15.38% neutral, and 34.62% negative. There was no statistically significant association ($\chi^2(6) = 3.0481$, $p = 0.803$), meaning HT levelled up attitudes as well, from the baseline.

However, perception was still significantly associated with education level ($\chi^2(6) = 13.5617$, $p = 0.035$). All postgraduates had positive or neutral perceptions, while those with primary education had exclusively negative perceptions. The majority of tertiary-level participants (65.38%) held positive perceptions.

Ethnicity and knowledge, attitude, and perception post-HT showed an evening out in the attitude and perception moieties.

Ewe participants exhibited the highest level of good knowledge (73.33%), followed by the Ga (66.67%) and Northern Ghanaian (60.00%) groups. Akan participants had a lower good knowledge rate (37.50%), and the overall association between ethnicity and

knowledge remained not statistically significant ($\chi^2(8) = 8.7651$, $p = 0.362$).

Attitude and perception (unlike at baseline) did not differ significantly across ethnic groups (attitude: $\chi^2(8) = 2.5270$, $p = 0.960$; perception: $\chi^2(8) = 2.1933$, $p = 0.975$). Most groups reported a high proportion of positive attitudes and perceptions, especially among Ewe and Northern Ghanaian participants.

Residence and knowledge, attitude, and perception post-HT, showed a levelling out in all the KAP components post-intervention.

Knowledge was high across all residence categories, with rural participants showing 65% good knowledge, followed by urban (59.26%) and suburban (58.33%). Unlike at baseline, no significant difference was observed ($\chi^2(4) = 2.4627$, $p = 0.651$).

Attitudes were most positive among suburban dwellers (66.67%), followed by urban (55.56%) and rural (40.00%) participants, although not statistically significant ($\chi^2(4) = 4.3929$, $p = 0.355$), unlike at baseline. Similarly, perception was highest in the suburban group (75.00%), followed by urban (62.96%) and rural (55.00%), with no significant association observed ($\chi^2(4) = 1.5490$, $p = 0.818$), unlike at baseline.

Family history of prostate cancer (FMH) post-HT showed mixed results.

There was still no significant association between FMH and knowledge ($\chi^2(2) = 0.0679$, $p = 0.967$), with 66.67% of those with FMH and 62.07% of those without FMH reporting good knowledge.

However, attitude and perception were significantly associated with PCa FMH. Among those with FMH, only 33.33% had a positive attitude compared to 55.17% of those without ($\chi^2(2) = 8.1854$, $p = 0.017$), similar to baseline. Additionally, 66.67% of those with FMH had a positive perception, compared to 63.79% of those without FMH ($\chi^2(2) = 9.7307$, $p = 0.008$), suggesting greater disparities in perception post-talk among participants with PCa FMH.

Lifestyle factors (tobacco, alcohol, exercise, and diet) post-HT all experienced an evening out, suggesting a bridge in disparities by the HT across the various groups studied.

Tobacco use did not show any statistically significant relationship (unlike at baseline) with knowledge ($\chi^2(2) = 0.6154$, $p = 0.735$), attitude ($\chi^2(2) = 0.8626$, $p = 0.650$), or perception ($\chi^2(2) = 2.0842$, $p = 0.353$). Former users and never-users had comparable proportions of positive views.

Alcohol use also showed no significant association with knowledge ($\chi^2(2) = 2.3787$, $p = 0.304$), attitude ($\chi^2(2) = 0.7925$, $p = 0.673$), or perception ($\chi^2(2) = 3.7983$, $p = 0.150$), unlike at baseline. However, those who never drank were slightly more likely to exhibit good knowledge and positive perceptions.

Unlike at baseline, exercise frequency was not significantly associated with any of the three outcome measures (knowledge: $\chi^2(6) = 3.6731$, $p = 0.721$; attitude: $\chi^2(6) = 8.3929$, $p = 0.211$; perception: $\chi^2(6) = 3.8248$, $p = 0.700$). Positive attitudes were most common among participants engaging in light exercise (72.73%).

Diet type also showed no significant associations (knowledge: $\chi^2(4) = 0.2549$, $p = 0.993$; attitude: $\chi^2(4) = 2.4807$, $p = 0.648$; perception: $\chi^2(4) = 0.4167$, $p = 0.981$), unlike at baseline, though vegetarians had consistently higher levels of good knowledge and positive attitudes. Figures *S5* A to C and S6 showcase some of these post-interventional shifts.

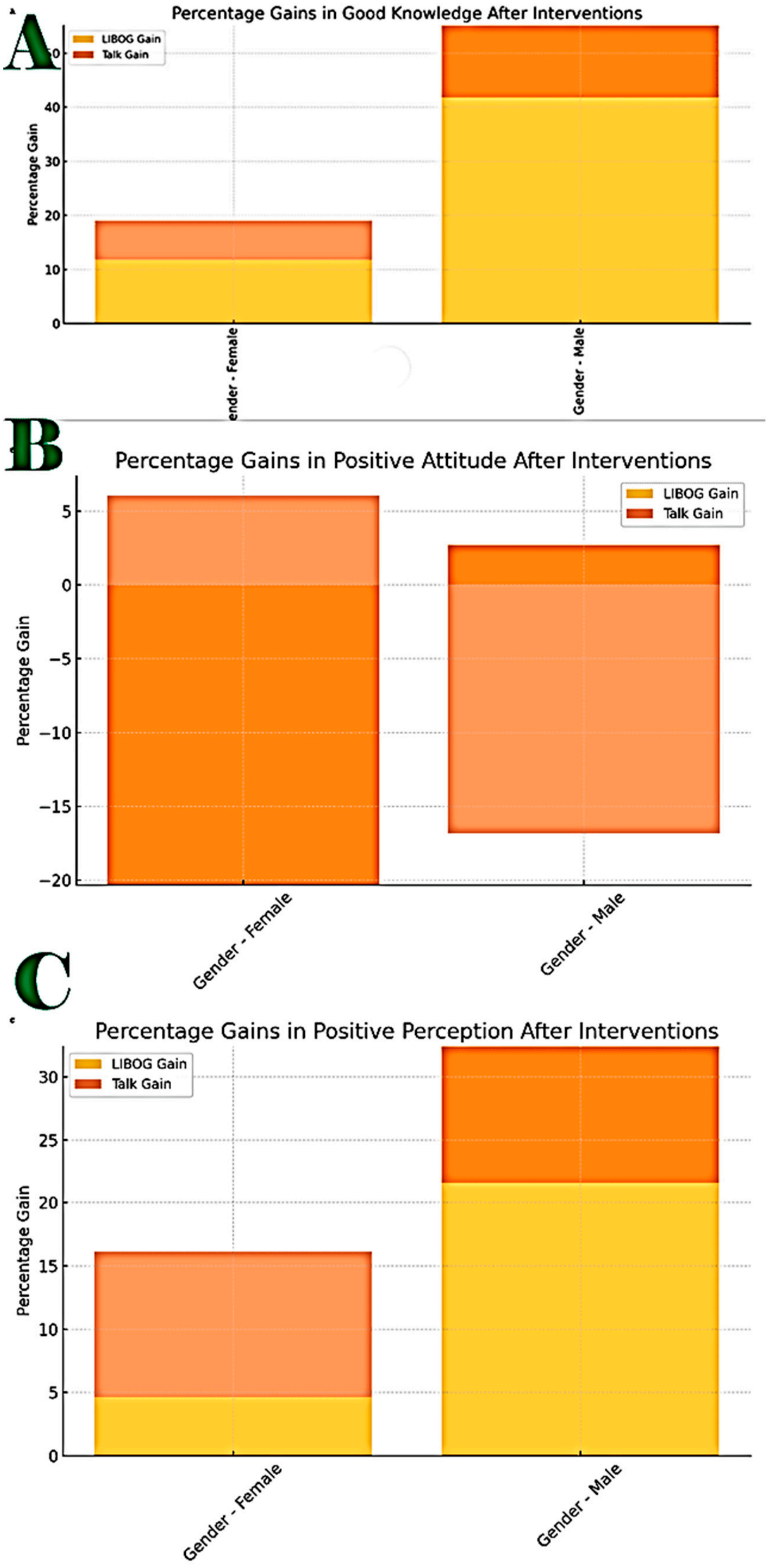
A
Percentage Gains in Good Knowledge After Interventions
LIBOG Gain
Talk Gain
Percentage Gain
0
10
20
30
40
ender - Female
Gender - Male
B
Percentage Gains in Positive Attitude After Interventions
LIBOG Gain
Talk Gain
Percentage Gain
5
0
−5
−10
−15
−20
Gender - Female
Gender - Male
C
Percentage Gains in Positive Perception After Interventions
LIBOG Gain
Talk Gain
Percentage Gain
0
5
10
15
20
25
30
Gender - Female
Gender - Male

**Figure S5.** Percentage changes in good knowledge, positive attitude, and positive perceptions after interventions with LIBOG or health talk (HT), disaggregated by gender. FIGURE LEGEND: Figure 8A shows the percentage gains in good knowledge among males and females following interventions. Both the LIBOG game and the standard health talk led to knowledge improvement, with higher gains seen in males. Figure 8B displays changes in positive attitude. Overall, the talk led to a slight positive shift in females; however, a reduction in attitude scores was observed following the LIBOG intervention, especially among males. Figure 8C illustrates percentage gains in positive perception, where males again showed a stronger improvement, particularly following the LIBOG intervention. Abbreviations: LIBOG—literacy board game (a participatory health education tool); HT—health talk (standard didactic health education session).

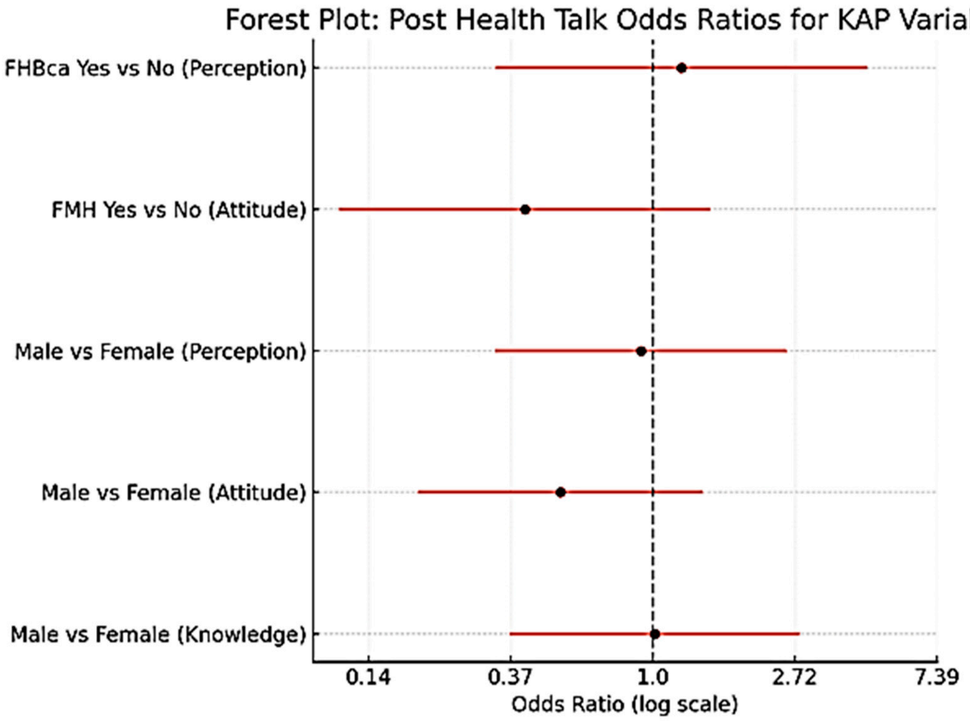


**Figure S6. Post health talk intervention forest plots for KAP odds ratios.** FIGURE LEGEND: None of the odds ratios shown remained statistically significant post-talk intervention.

Regression analysis post-HT: Following the prostate cancer health talk (intervention 2), several odds ratio analyses were conducted to examine relationships between key demographic and familial variables and the likelihood of demonstrating good knowledge, a positive attitude, or a positive perception post-HT.

In terms of knowledge, the odds of having good knowledge among males were virtually identical to those among females, with an odds ratio of 1.01 (95% CI: 0.36 to 2.81, p = 0.982). This indicates no significant gender-based difference in knowledge acquisition, and a levelling up after the intervention.

When examining attitude, males were less likely than females to have a positive attitude, with an odds ratio of 0.52 (95% CI: 0.19 to 1.42, p = 0.204). However, this difference was not statistically significant. Similarly, the odds of having a positive perception were slightly lower among males compared to females, with an odds ratio of 0.92 (95% CI: 0.33 to 2.57, p = 0.873), again showing no significant difference (bridged disparities after HT exposure).

Turning to family history of prostate cancer, participants with such a history had lower odds of expressing a positive attitude than those without, with an odds ratio of 0.41 (95% CI: 0.11 to 1.50, p = 0.177). While suggestive of a trend, this finding did not reach statistical significance. For perception, individuals with a family history of prostate cancer had marginally higher odds of expressing a positive perception compared to those without such history, with an odds ratio of 1.22 (95% CI: 0.33 to 4.54, p = 0.764); however, this too was not statistically significant.

Overall, these results suggest that the educational intervention had a broadly equalizing effect across gender and family history subgroups, with no significant disparities observed in knowledge, attitude, or perception outcomes.

Differences in Knowledge Attitude and Perception between Baseline and Final for Both LIBOG and Health Talk:

The final output table presents a comparative analysis of baseline and post-intervention performances across three outcome domains—knowledge, attitude, and perception—following two different educational interventions: a standard health talk (HT) and an interactive LIBOG game. It organizes the metrics into statistical descriptors (mean, median, mode, standard deviation, range) and tracks the percentage of participants with good and poor outcomes before and after interventions. It further includes absolute changes from baseline (improvements), relative performance of LIBOG vs talk, and tests of statistical significance (Z-tests and p-values) (see Tables S4, S *5*, S*6*, S*7*; Figures *S7 A* and B).

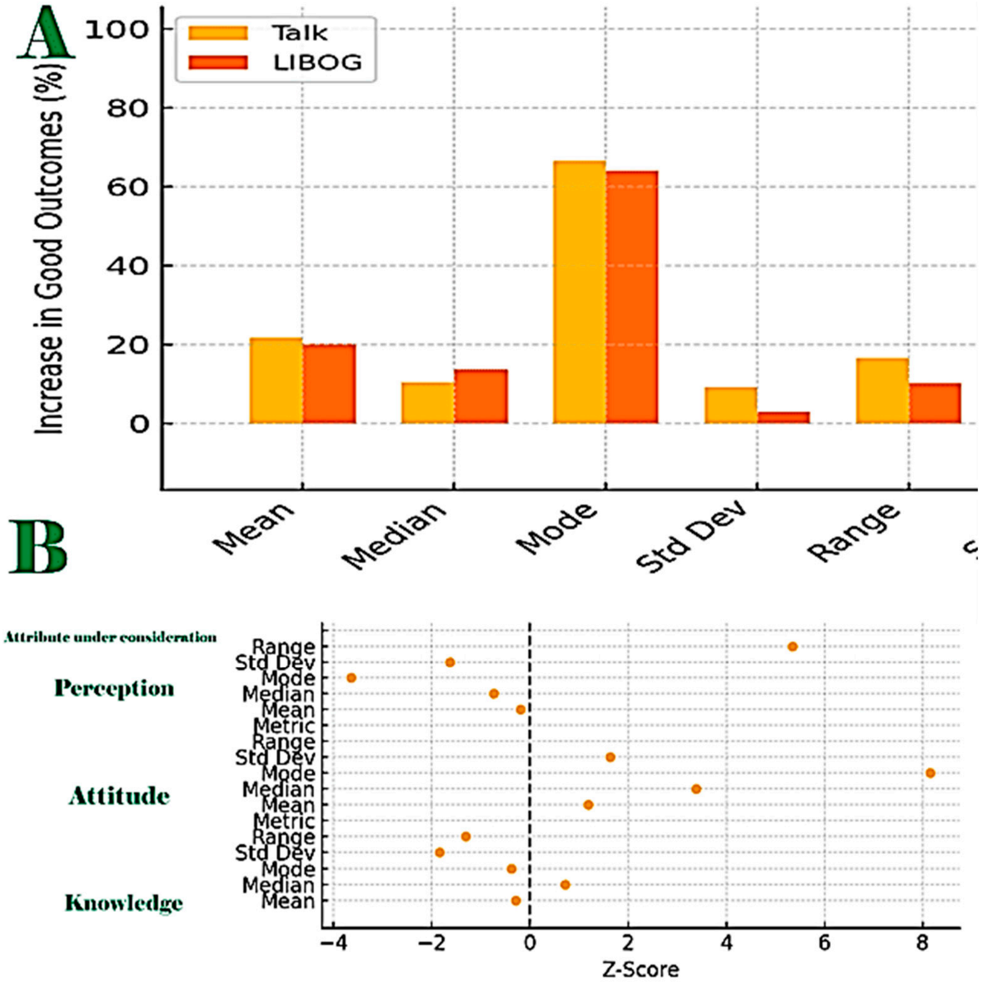


**Figure S7.** Bar chart showing comparative gains in knowledge, attitude, and perception (altogether)—LIBOG vs health talk and forest plot. Statistical significance of improvement differences between LIBOG and talk. FIGURE LEGEND: A: The bar chart displays the percentage increase in good outcomes from baseline for each health literacy domain—knowledge, attitude, and perception (altogether)—stratified by statistical metrics (mean, median, mode, range, standard deviation). For each metric, the LIBOG and talk interventions are plotted. It is observed that for the mean, median and mode, the performance of the two interventions are nearly neck-and-neck. Also, the fact that the range and standard deviational changes are smaller for LIBOG may suggest that the HT surpasses in narrowing disparities in 'good outcomes'. B: The forest plot represents Z-test statistics comparing the gains made by LIBOG vs the talk intervention across all metrics. Each dot corresponds to a Z-score for a metric, indicating whether the LIBOG game was significantly more effective (positive values) or not. The vertical dashed line ($Z = 0$) represents equivalence. Statistically significant points ($Z > \pm1.96$) occur for modal attitude ($z = 3.85$, $p < 0.001$) and attitudinal range ($z = -2.07$, $p = 0.038$), demonstrating superiority of LIBOG in these domains.

**Table S4.** Comparatives for the measures of central tendencies (mean, median, mode) and measures of dispersion (range and standard deviation) for all KAP baseline values and KAP changes across all the 14 socio-demographic and lifestyle factors studied after LIBOG and HT interventions.

| **Metric** | **--- KNOWLEDGE ---** | **Mean** | **Median** | **Mode** | **Std Dev** | **Range** |
|---|---|---|---|---|---|---|
| Baseline_Good knowledge% | | 41.86 | 51.52 | 0 | 20.75 | 66.67 |
| Baseline_Poor knowledge% | | 53.1 | 41.54 | 100 | 22.29 | 72.73 |
| PostTalk_Good knowledge% | | 63.75 | 62.07 | 66.67 | 11.41 | 50 |
| PostTalk_Poor knowledge% | | 7.31 | 0 | 0 | 15.13 | 37.5 |
| Increase_Good knowledge levels_Talk | | 21.88 | 10.55 | 66.67 | 9.34 | 16.67 |
| Decrease_Poor knowledge levels_Talk | | 45.79 | 41.54 | 100 | 7.16 | 35.23 |
| Significant_Increase_Good knowledge levels_Talk | | z=3.10, p=0.002 | z=1.51, p=0.132 | z=10.00, p=0.000 | z=-1.80, p=0.072 | z=-2.39, p=0.017 |
| Significant_Decrease_Poor knowledge levels_Talk | | z=7.05, p=0.000 | z=7.24, p=0.000 | z=14.14, p=0.000 | z=1.30, p=0.194 | z=5.01, p=0.000 |
| Post-LIBOG_Good knowledge% | | 62 | 65.38 | 64.1 | 17.51 | 56.35 |
| Post-LIBOG_Poor knowledge% | | 38 | 34.62 | 35.9 | 17.51 | 56.35 |
| Increase_Good knowledge level_LIBOG | | 20.2 | 13.86 | 64.1 | 3.04 | 10.32 |
| Decrease_Poor knowledge levels_LIBOG | | 15.1 | 7.12 | 64.1 | 19.95 | 16.38 |
| Significant_Increase_Good knowledge_LIBOG | | z=2.85, p=0.004 | z=1.99, p=0.047 | z=9.71, p=0.000 | z=-0.58, p=0.560 | z=-1.50, p=0.134 |
| Significant_Decrease_Poor knowledge_LIBOG | | z=2.14, p=0.032 | z=1.01, p=0.314 | z=9.71, p=0.000 | z=0.85, p=0.397 | z=2.42, p=0.015 |
| Relative_Improvements; LIBOG/TALK | | 0.3 | 0.23 | 0.62 | 0.91 | 0.29 |
| Significant_Difference in Improvements; LIBOG vs TALK | | z=-0.29, p=0.771 | z=0.72, p=0.475 | z=-0.38, p=0.702 | z=-1.85, p=0.065 | z=-1.31, p=0.189 |
| Metric | --- ATTITUDE --- | Mean | Median | Mode | Std Dev | Range |
| Baseline_Good Attitude% | | 43.37 | 54.67 | 0 | 21.3 | 66.67 |
| Baseline_Poor Attitude% | | 43.07 | 28.57 | 100 | 28.75 | 100 |
| PostTalk_Good Attitude% | | 54.83 | 53.33 | 50 | 16.67 | 71.43 |
| PostTalk_Poor Attitude% | | 7.42 | 0 | 0 | 16 | 50 |
| Increase_Good Attitude levels_Talk | | 11.46 | -1.34 | 50 | 4.63 | 4.76 |
| Decrease_Poor Attitude levels_Talk | | 35.65 | 28.57 | 100 | 12.75 | 50 |
| Significant_Increase_Good Attitude levels_Talk | | z=1.62, p=0.105 | z=-0.19, p=0.849 | z=8.16, p=0.000 | z=-0.83, p=0.404 | z=0.73, p=0.467 |
| Significant_Decrease_Poor Attitude levels_Talk | | z=5.80, p=0.000 | z=5.77, p=0.000 | z=14.14, p=0.000 | z=2.16, p=0.031 | z=8.16, p=0.000 |
| Post-LIBOG_Good Attitude% | | 60.75 | 60 | 100 | 32.09 | 77.78 |
| Post-LIBOG_Poor Attitude% | | 39.25 | 40 | 0 | 32.09 | 77.78 |
| Increase_Good Attitude level_LIBOG | | 17.38 | 5.37 | 100 | 10.79 | -11.11 |
| Decrease_Poor Attitude levels_LIBOG | | 3.82 | -12.43 | 100 | 3.3 | 22.22 |
| Significant_Increase_Good Attitude_LIBOG | | z=2.46, p=0.014 | z=0.76, p=0.446 | z=14.14, p=0.000 | z=1.72, p=0.085 | z=1.75, p=0.079 |
| Significant_Decrease_Poor Attitude_LIBOG | | z=0.55, p=0.583 | z=-1.70, p=0.089 | z=14.14, p=0.000 | z=-0.51, p=0.608 | z=5.00, p=0.000 |

| | | | | | | |
|---|---|---|---|---|---|---|
| Relative_Improvements; LIBOG/TALK | | 0.16 | 1.74 | 2 | 0.6 | -1.04 |
| Significant_Difference in Improvements; LIBOG vs TALK | | z=1.19, p=0.233 | z=3.38, p=0.001 | z=8.16, p=0.000 | z=1.63, p=0.102 | |
| Metric | --- PERCEPTION --- | Mean | Median | Mode | Std Dev | Range |
| Baseline_Good Perception% | | 35.38 | 40.95 | 0 | 21.33 | 100 |
| Baseline_Poor Perception% | | 38.44 | 44.42 | 0 | 20.12 | 100 |
| PostTalk_Good Perception% | | 58.59 | 64.29 | 50 | 17.43 | 71.43 |
| PostTalk_Poor Perception% | | 13.15 | 0 | 0 | 27.47 | 28.57 |
| Increase_Good Perception levels_Talk | | 23.21 | 23.34 | 50 | 3.9 | 0 |
| Decrease_Poor Perception levels_Talk | | 25.29 | 44.42 | 0 | 5.35 | 0 |
| Significant_Increase_Good Perception levels_Talk | | z=3.29, p=0.001 | z=3.31, p=0.001 | z=8.16, p=0.000 | z=-0.70, p=0.485 | z=-5.77, p=0.000 |
| Significant_Decrease_Poor Perception levels_Talk | | z=4.09, p=0.000 | z=7.56, p=0.000 | z=0.00, p=1.000 | z=-1.22, p=0.222 | z=10.54, p=0.000 |
| Post-LIBOG_Good Perception% | | 57.48 | 60 | 25 | 21.84 | 75 |
| Post-LIBOG_Poor Perception% | | 42.52 | 40 | 0 | 21.84 | 75 |
| Increase_Good Perception level_LIBOG | | 22.1 | 19.05 | 25 | 0.51 | 25 |
| Decrease_Poor Perception levels_LIBOG | | 7.18 | -4.42 | 0 | -1.72 | 25 |
| Significant_Increase_Good Perception_LIBOG | | z=3.13, p=0.002 | z=2.69, p=0.007 | z=5.35, p=0.000 | z=0.09, p=0.930 | z=-5.35, p=0.000 |
| Significant_Decrease_Poor Perception_LIBOG | | z=-0.59, p=0.557 | z=0.63, p=0.527 | z=0.00, p=1.000 | z=-0.30, p=0.765 | z=5.35, p=0.000 |
| Relative_Improvements; LIBOG/TALK | | 0.27 | -0.08 | | -0.04 | |
| Significant_Difference in Improvements; LIBOG vs TALK | | z=-0.19, p=0.851 | z=-0.74, p=0.458 | z=-3.65, p=0.000 | z=-1.63, p=0.103 | z=5.35, p=0.000 |

TABLE LEGEND: HT = health talk. LIBOG = literacy board game.

**Table S5.** Knowledge domain: baseline, post-LIBOG and post-HT; mean, median, mode, range, standard deviation and statistically significant differences.

| Knowledge Aggregate Metric | Baseline Good (%) | Post-Talk Good (%) | Post-LIBOG Good (%) | LIBOG Gain (%) | Talk Gain (%) | Relative Improvement LIBOG/HT | LIBOG vs Talk Z-test |
|---|---|---|---|---|---|---|---|
| Mean | 41.86 | 63.75 | 62.00 | 20.2 | 21.88 | 0.92 | z = –0.29, p = 0.771 |
| Median | 51.52 | 62.07 | 65.38 | 13.86 | 10.55 | 1.31 | z = 0.72, p = 0.475 |
| Mode | 0.00 | 66.67 | 64.10 | 64.1 | 66.67 | 0.96 | z = –0.38, p = 0.702 |
| Std Dev | 20.75 | 11.41 | 17.51 | 3.04 | 9.34 | 0.33 | z = –1.85, p = 0.065 |

|  |  |  |  |  |  |  |  |
|---|---|---|---|---|---|---|---|
| Range | 66.67 | 50.00 | 56.35 | 10.32 | 16.67 | 0.62 | z = −1.31, p = 0.189 |

TABLE LEGEND: HT = health talk. LIBOG = literacy board game.

**Table S6.** Attitude domain: baseline, post-LIBOG and post-HT; mean, median, mode, range, standard deviation and statistically significant differences.

| Attitude Aggregate Metric | Baseline Good (%) | Post-Talk Good (%) | Post-LIBOG Good (%) | LIBOG Gain (%) | Talk Gain (%) | Relative Improvement | LIBOG vs Talk Z-test |
|---|---|---|---|---|---|---|---|
| Mean | 43.37 | 54.83 | 60.75 | 17.38 | 11.46 | 0.53 | z = 1.28, p = 0.199 |
| Median | 54.67 | 53.33 | 60.00 | 5.37 | −1.34 | Undefined | z = 1.57, p = 0.115 |
| Mode | 0.00 | 50.00 | 100.00 | 100.0 | 50.0 | 2.0 | z = 3.85, p = 0.000 |
| Std Dev | 21.30 | 16.67 | 32.09 | 10.79 | 4.63 | 2.33 | z = 0.95, p = 0.343 |
| Range | 66.67 | 71.43 | 77.78 | −11.11 | 4.76 | Negative | z = −2.07, p = 0.038 |

TABLE LEGEND: HT = health talk. LIBOG = literacy board game.

**Table S7.** Perception domain: baseline, post-LIBOG and post-HT; mean, median, mode, range, standard deviation and statistically significant differences.

| Perception Aggregate Metric | Baseline Good (%) | Post-Talk Good (%) | Post-LIBOG Good (%) | LIBOG Gain (%) | Talk Gain (%) | Relative Improvement | LIBOG vs Talk Z-test |
|---|---|---|---|---|---|---|---|
| Mean | 35.38 | 57.48 | 58.59 | 25.29 | 7.18 | 0.53 | z = -0.19, p = 0.851 |
| Median | 40.95 | 60 | 64.29 | 44.42 | 19.05 | 0.57 | z = -0.74, p = 0.458 |
| Mode | 0 | 25 | 50 | 50 | 25 | 1 | z = -3.65, p = 0.000 |
| Standard Deviation | 21.33 | 21.84 | 17.43 | -3.9 | 0.51 | -7.65 | z = -1.63, p = 0.103 |
| Range | 100 | 75 | 71.43 | -28.57 | -25 | -0.14 | z = 5.35, p = 0.000 |

TABLE LEGEND: HT = health talk. LIBOG = literacy board game.

Key Insight: Both interventions significantly improved good knowledge outcomes (Tables 5 and 6). LIBOG and talk showed comparable effectiveness overall, with health talk slightly edging LIBOG in mean gain but LIBOG showing a steadier median and smaller variability. However, no statistically significant difference was found between the LIBOG and talk improvements (all $p > 0.05$). The two are comparable. LIBOG compared favorably with HT in the knowledge moiety.

Key Insight: The LIBOG game led to larger gains in attitude (Tables S4 and S5), especially in mode and mean values, with a statistically significant difference observed for the modal value ($z = 3.85$, $p < 0.001$). The range analysis suggests LIBOG also reached more individuals at the extremes (better outcomes for some), despite slightly higher variability. LIBOG may be superior in modifying the attitude moiety.

Perception Domain: baseline, post-LIBOG and post-HT; mean, median, mode, range, standard deviation and statistically significant differences.

Perception metrics were also analyzed separately. The LIBOG intervention demonstrated positive perception shifts that outperformed the talk intervention (Table S4).

*S3.12. Comparison by Aggregates*

Aggregates of HT gains: The health talk intervention yielded notable improvements in both positive and negative KAP attributes across all three domains:

Knowledge: The total improvement in good knowledge levels was 125.11%, averaging 25.02% across the five statistical measures.

Reduction in poor knowledge level: An even more substantial improvement was observed here, with a total of 229.72%, averaging 45.94%.

The overall (final) aggregate score was 354.83%, translating to an average of 35.48% improvement in the knowledge domain.

Attitude:

Improvement in Positive Attitude: Total = 69.51%, average = 13.90%.

Reduction in Negative Attitude: Total = 226.97%, average = 45.39%.

Final Aggregate: Total = 296.48%, average = 29.64%.

Perception:

Improvement in Positive perception: Total = 100.45%, average = 20.09%.

Reduction in Negative perception: Total = 75.01%, average = 15.00%.

Final Aggregate: Total = 175.46%, average = 17.55%.

*S3.13. Overall (Grand Totals) for Health Talk*

Positive Improvements: Total = 295.07% (average = 19.67%).

Negative Reductions: Total = 531.70% (average = 35.45%).

The combined grand total was 826.79%, yielding a grand average of 27.56% across all KAP domains and demographic strata (Table S8).

**Table S8.** Table for intervention by health talk; aggregation of percentage improvements across 5 measures of central tendency and dispersion (mean, median, mode, range, standard deviation), across all three KAPS over the 14 socio-demographic parameters studied.

| Attribute Under Consideration | Improvement in Positive Attributes % | Reduction in Negative Attributes % | Final Totals and Averages% |
|---|---|---|---|
| Knowledge | Total = 125.11, Average= 25.02% | Total = 229.72, Average=45.94% | (354.83; 35.48%) |
| Attitude | Total = 69.51, Average= 13.90% | Total = 226.97, Average= 45.39% | (296.48; 29.64%) |
| Perception | Total = 100.45, Average= 20.09% | Total = 75.01, Average= 15.00% | 175.46; 17.55% |
| Grand Total and Grand Average | (295.07; 19.67%) | (531.70; 35.45%) | (826.79; 27.56%) |

Aggregates for LIBOG Game Intervention:

The LIBOG board game intervention, which emphasizes interactivity and participation, also demonstrated strong gains, especially in attitude and perception:

Knowledge:

Improvement in Positive knowledge: Total = 111.52%, average = 22.30%.

Reduction in Negative knowledge: Total = 122.65%, average = 24.53%.

Final Aggregate: Total = 234.17%, average = 23.47%.

Attitude:

Improvement in Positive Attitude: Total = 122.43%, average = 24.49%.

Reduction in Negative Attitude: Total = 116.91%, average = 23.38%.

Final Aggregate: Total = 239.34%, average = 23.93% (Table S9).

**Table S9.** Table for intervention by LIBOG aggregation of percentage improvements across 5 measures of central tendency and dispersion (mean, median, mode, range, standard deviation), across all three KAPS, over the 14 socio-demographic parameters studied.

| Attribute Under Consideration | Improvement in Positive Attributes % | Reduction in Negative Attributes % | Final Totals, and Averages% |
|---|---|---|---|
| Knowledge | Total = 111.52, Average= 22.30% | Total = 122.65, Average= 24.53% | (234.17; 23.47%) |
| Attitude | Total = 122.43, Average= 24.49% | Total = 116.91, Average= 23.38% | (239.34; 23.93%) |
| Perception | Total = 91.66, Average= 18.33 | Total = 26.04, Average= 5.21% | (117.0; 11.77%) |
| Grand Total and Grand Average | (325.61; 21.70%) | (262.94; 17.53%) | (588.56; 19.61%) |

Perception:

Improvement in Positive perception: Total = 91.66%, average = 18.33%.

Reduction in Negative perception: Total = 26.04%, average = 5.21%.

Final Aggregate: Total = 117.0%, average = 11.77%.

Overall (Grand Totals) for LIBOG Game

Positive Improvements: Total = 325.61%, average = 21.70%.

Negative Reductions: Total = 262.94%, average = 17.53%.

The combined grand total was 588.56%, with a grand average of 19.61% across all domains and parameters.

**Table S 10.** Comparative insights for LIBOG vs. HT.

| Dimension | Health Talk Avg. (%) | LIBOG Avg. (%) | Difference |
|---|---|---|---|
| Knowledge | 35.48 | 23.47 | HT leads by 12.01 pts |
| Attitude | 29.64 | 23.93 | HT leads by 5.71 pts |
| Perception | 17.55 | 11.77 | HT leads by 5.78 pts |
| Grand Avg. | 27.56 | 19.61(71% of HT's) | HT leads by 7.95 pts |

TABLE LEGEND: HT = health talk. LIBOG = literacy board game.

While both interventions were effective, the health talk consistently produced higher percentage improvements and reductions, especially in knowledge and attitude domains. However, the LIBOG game still showed appreciable gains, particularly in affective and perceptual areas, and may offer benefits in engagement and longer-term behavioral reinforcement.

In the final analysis, HT leads by 7.95% points overall ahead of LIBOG, but is this difference statistically significant?

Statistical Test: Difference Between Proportions

To assess whether the observed difference in overall grand average performance between the health talk (HT) group and the LIBOG group was statistically significant, a Z-test for two proportions was performed.

Input Values:

Health Talk Mean ($p_1$): 27.56% → 0.2756

LIBOG Mean ($p_2$): 19.61% → 0.1961

Sample Size (HT): 61

Sample Size (LIBOG): 80

Difference in Means: 7.95 percentage points

Test Output: Z-score = 1.11, p-value = 0.267

Interpretation:

The observed advantage of the health talk group (+7.95%) over the LIBOG group is not statistically significant at the conventional 5% threshold ($p = 0.267$). While numerically higher, the difference in average overall gains could plausibly be due to random variation given the sample sizes involved.

Finally, to cross-check, the Wilcoxon Signed-Rank Test was conducted, with the objective of determining whether there was a statistically significant difference (in medians) between the effectiveness (in percentage gain) of two paired educational interventions: LIBOG and standard health talk.

The test was conducted on the assumptions that each pair of values represents the percentage gain in knowledge, attitude, or perception for the same subgroup across both interventions. It was based on the following assumptions:

The data are paired and come from the same population. The paired differences are continuous and symmetrically distributed around the median. The dependent variable (here, percentage gain) is ordinal or continuous and the observations are mutually independent.

The null hypothesis ($H_0$) that the test was based on stated the following:

There is no difference in median percentage gains between LIBOG and health talk.

$H_0$: median (LIBOG - Talk) = 0

The test was conducted to involve all 14 sociodemographic parameters, over both sets as follows:

Good and poor knowledge gains, good and poor attitude gains, and good and poor perception gains across 25 subgroup comparisons. The test results were as follows:

Test Statistic (W) = 102.0, p-value = 0.107.

Interpretation: Since $p = 0.107 > 0.05$, we failed to reject the null hypothesis.

There is no statistically significant difference in the overall effectiveness of the LIBOG vs the health talk when combining all domains and subgroup improvements (Figure S8).

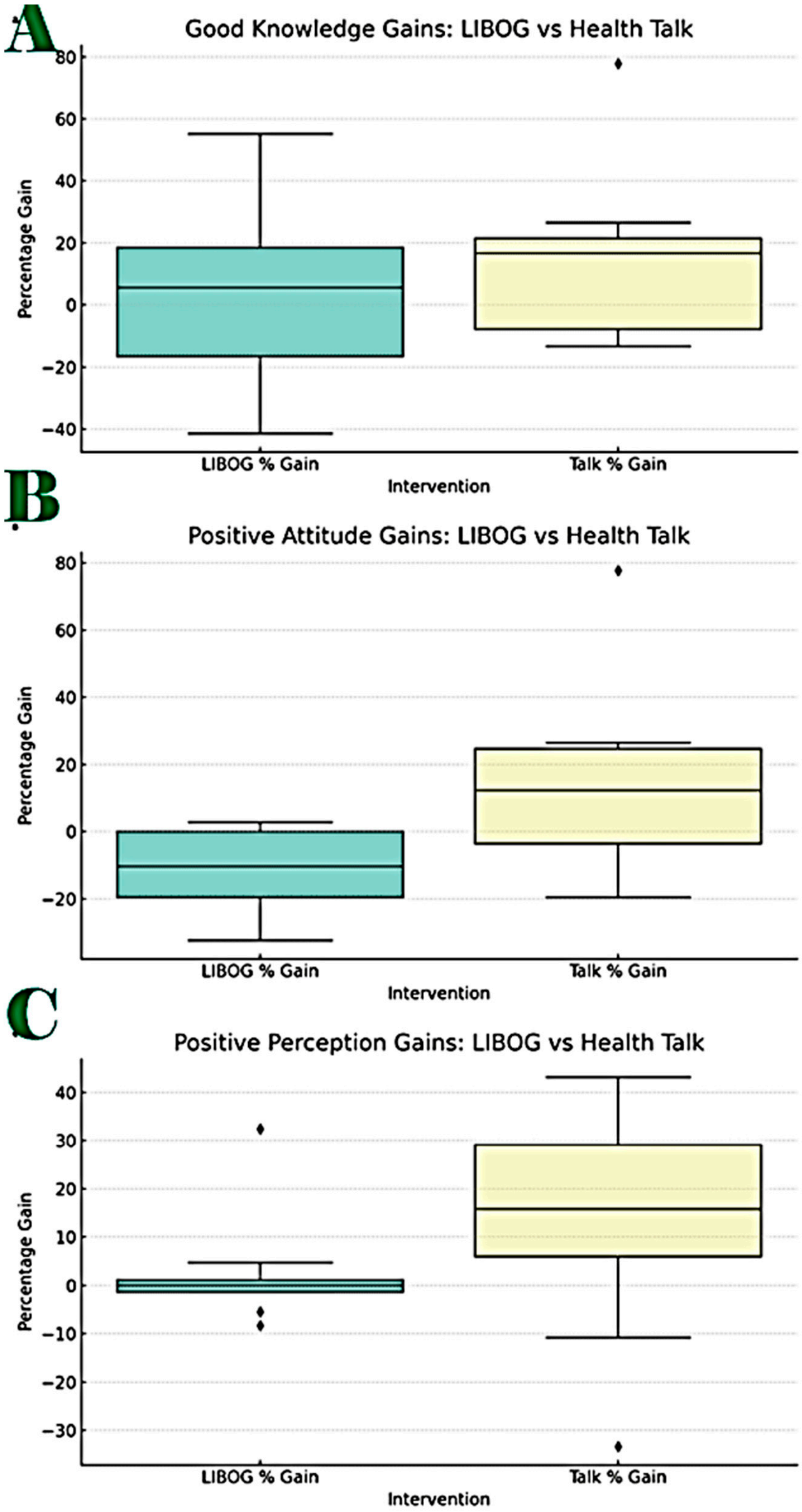


**Figure S8.** Box plots for the Wilcoxon Signed-Rank Test analysis: LIBOG vs. HT. FIGURE LEGEND: Good knowledge: number of paired comparisons: 9, Wilcoxon Signed-Rank Statistic (W): 18.0, p-value: 0.6523. Result: no

statistically significant difference (Figure 11). Positive attitude: number of paired comparisons: 8, Wilcoxon Signed-Rank Statistic (W): 5.0, p-value: 0.0781. Result: no statistically significant difference. Positive perception: Number of paired comparisons: 8, Wilcoxon Signed-Rank Statistic (W): 12.0, p-value: 0.4609. Result: no statistically significant difference.

**S14 Important Resources:**

1. The drive for the prostate cancer S-S-LIBOG and its manuals can be found at the following links:

THE 18 LIBOGS (jpegs)

https://drive.google.com/file/d/1Z3i-SjaH4jeP172rPvGnweWRHnuktdSR/view?usp=sharing

THE 18 LIBOGS (PDF): These include S-S-LIBOGs on adolescent sexual and reproductive health, asthma, antimicrobial resistance, breast cancer, breastfeeding/Breastfeeding Hospital Initiative, cervical cancer, diabetes mellitus, hypertension, family planning, hematuria and bladder cancer, kidney diseases, male circumcision complications, malnutrition (in children), mental health, menstrual health, prostate cancer, sickle cell disease.

https://drive.google.com/file/d/1YZB7CSeCmGruRSp25OXMxZj6dGPul5j4/view?usp=sharing

THE MANUALS FOR THE 18 LIBOGS

https://drive.google.com/drive/folders/1KMSfB9k4JNNjox9f92TC6Kaj09daVYdV?usp=sharing

2. The prototype digital version of the LIBOG is available at the following link:

https://derrik1-dev.github.io/ludolite/doit.html

3. The standard PowerPoint slides used for the HT are available at the following link:

https://drive.google.com/file/d/1HDkq6X6gwL_f82CErxZhxuhBXXKoEYoW/view?usp=sharing

4. Health talk guides for the other 16 diseases.

https://drive.google.com/file/d/1Av1jQ4t_8ZbPxVGgRgRI6SnG4kUMFZRB/view?usp=sharing
